# Nonlinear Optical Pigments. Two-Photon Absorption in Crosslinked Conjugated Polymers and Prospects for Remote Nonlinear Optical Thermometry

**DOI:** 10.3390/polym12081670

**Published:** 2020-07-27

**Authors:** Jan K. Zaręba, Marcin Nyk, Marek Samoć

**Affiliations:** Advanced Materials Engineering and Modelling Group, Wroclaw University of Science and Technology, Wyb. Wyspiańskiego 27, 50-370 Wrocław, Poland; marcin.nyk@pwr.edu.pl (M.N.); marek.samoc@pwr.edu.pl (M.S.)

**Keywords:** nonlinear optics, two-photon absorption, nonlinear optical pigments, two-photon excited fluorescence, non-contact luminescent probes, temperature-responsive materials

## Abstract

Nonlinear optical (NLO) pigments are compounds insoluble in solvents that exhibit phenomena related to nonlinear optical susceptibilities (χ^(n)^ where n = 2,3,...), e.g., two-photon absorption (2PA) which is related to the imaginary part of χ^(3)^. Determination of spectrally-resolved 2PA properties for NLO pigments of macromolecular nature, such as coordination polymers or crosslinked polymers, has long been a challenging issue due to their particulate form, precluding characterizations with standard techniques such as Z-scan. In this contribution, we investigate thus far unknown spectrally-resolved 2PA properties of a new subclass of NLO pigments—crosslinked conjugated polymers. The studied compounds are built up from electron-donating (triphenylamine) and electron-withdrawing (2,2’-bipyridine) structural fragments joined by vinylene (**Pol1**) or vinyl(4-ethynylphenyl) (**Pol2**) aromatic bridges. 2PA properties of these polymers have been characterized in broad spectral range by specially modified two-photon excited fluorescence (TPEF) techniques: solid state TPEF (SSTPEF) and internal standard TPEF (ISTPEF). The impact of self-aggregation of aromatic backbones on the 2PA properties of the polymers has been evaluated through extended comparisons of NLO parameters, i.e., 2PA cross sections (σ_2_) and molar-mass normalized 2PA merit factors (σ_2_/M) with those of small-molecular model compounds: **Mod1** and **Mod2**. By doing this, we found that the 2PA response of **Pol1** and **Pol2** is improved 2–3 times versus respective model compounds in the solid state form. Further comparisons with 2PA results collected for diluted solutions of **Mod1** and **Mod2** supports the notion that self-aggregated structure contributes to the observed enhancement of 2PA response. On the other hand, it is clear that **Pol1** and **Pol2** suffer from aggregation-caused quenching phenomenon, well reflected in time-resolved fluorescence properties as well as in relatively low values of quantum yield of fluorescence. Accordingly, despite improved intrinsic 2PA response, the effective intensity of two-photon excited emission for **Pol1** and **Pol2** is slightly lower relative to **Mod1** and **Mod2**. Finally, we explore temperature-resolved luminescence properties under one- (377 nm), two- (820 nm), and three-photon excitation (1020 nm) conditions of postsynthetically Eu^3+^-functionalized material, **Pol1-Eu**, and discuss its suitability for temperature sensing applications.

## 1. Introduction

The interest in polymers as candidate materials for applications employing second-order and third-order nonlinear optical (NLO) phenomena dates back to the 1980s and has been maintained ever since [1,2]. This has been elicited by the promising optical and structural properties of polymeric materials, as well as their ease of modification, largely defined by their organic nature. For instance, unlike inorganic matter, organic polymers feature large non-resonant electronic nonlinearities originating from π-electron contributions, which can be relatively easily tuned via structural modifications of their backbone. At the same time, large optical nonlinearities are accompanied by temporal responses that can be as low as in the femtosecond range [3], raising hopes for applications in optical signal processing, e.g., in all-optical switching [4,5,6]. On the synthetic side, a considerable body of information has been accumulated about how to maximize NLO response of organic compounds and led to the determination of molecular design principles for organic [7,8,9,10] and organometallic NLO chromophores [11,12,13,14]. These principles are now commonplace tools that assist the design of new small-molecular and polymeric compounds for nonlinear optics. From the processing point of view, polymers and polymeric materials appear to be exceptionally versatile as well, as some of them can be prepared in the form of liquid solutions or dispersions but also can be readily processed as powders, fibers, and films, and some selected examples of them can be obtained as single crystals. Accordingly, favorable optical and physicochemical properties of polymers created a fertile niche for investigation of various aspects of their NLO properties, such as electro-optic phenomena, i.e., the linear electro-optic effect (Pockels effect) [15], the quadratic electro-optic effect (optical Kerr effect) [16,17,18], parametric energy conversion in second-harmonic [11,12,13,14,19,20,21] and third-harmonic generation [22,23,24,25,26] variants, and finally the two-photon absorption phenomenon (2PA) [26,27,28,29,30,31], which is at the focus of this paper. 

A majority of extant studies on 2PA properties of polymers has employed the Z-scan technique [32,33,34,35,36,37,38] and, to a lesser extent, the two-photon excited fluorescence (TPEF) [39,40,41,42] as optical characterization techniques. The former technique, in general, has grown to be the most frequently used method of the characterization of third-order (and higher-order) NLO properties of materials that are available in the form of solutions or that could be fabricated as amorphous, uniform films. A significant advantage of the Z-scan is that it allows for a relatively sensitive determination of the nonlinear absorption and refraction parameters without the need for an external 2PA standard. On the other hand, Z-scan is a transmission-based technique, in which light transmittance changes are correlated with the strength of nonlinear absorption, making its determination susceptible to any factors that affect the transmitted light intensity. It follows that polymer samples ought to fulfill a stringent requirement of high transparency and low scattering to yield Z-scan traces that would quantitatively reflect the occurring NLO processes, rather than light losses due to scattering. In turn, the TPEF technique in its most prevalent, relative variant rests on the measurement of TPEF signal from a solution of 2PA chromophore and that of a solution of a reference compound for which the dispersion of the 2PA cross sections is precisely known. The measurement relative to an external reference necessitates preservation of the same scattering conditions for sample and a reference; hence, proper determination of 2PA cross sections also requires a polymer solution to be non-scattering. As can be seen, there are fundamental limitations of Z-scan and TPEF techniques that hinder their use for polymeric materials that are available in the form of insoluble solids, e.g., due to extensive crosslinking. It is therefore not a coincidence that all extant 2PA studies on polymers explored NLO properties of only those polymers, whose structures were linear, or more generally, one-dimensional, since only polymers of that topology are easily soluble or processable into optically uniform films. 

Unsuitability of Z-scan and TPEF techniques for the characterization of scattering samples leaves us with the question of how to properly determine the 2PA cross sections of polymers that form multi-connected macromolecular networks, as is the case with crosslinked polymers. Indeed, from a broader perspective, this apparent gap in NLO characterization toolbox has not been limited to the class of crosslinked polymers, but was plaguing all macromolecular materials e.g., coordination polymers (CPs), causing their nonlinear absorption properties to remain essentially unknown for years. As a remedy to this issue, specially modified TPEF techniques i.e., Internal Standard TPEF (ISTPEF) and Solid State TPEF (SSTPEF) have been devised recently [43]. CPs and their subclass, metal-organic frameworks (MOFs), were the first materials characterized with those techniques [43,44,45,46]. Results collected thus far are highly promising, as they demonstrate that CPs and MOFs offer very good NLO responses, which are sufficiently high to realize photonic applications, for example two-photon pumped lasing [47] or non-contact luminescence thermometry mediated by three-photon absorption [48]. From the fundamental science point of view, it also emerged that the solid state, aggregated nature of those compounds itself creates new pathways for the enhancement of nonlinear absorption [49]. One of the postulated mechanisms that are seen responsible for enhanced NLO properties of CPs is the aggregation of aromatic ligands and the conformational strain connected with this [46]. Accordingly, the preparation of new compounds whose organic backbones aggregate in the solid state might appear as a new, promising way to engineer materials with boosted 2PA properties. Nevertheless, given that this area is still in its infancy, much more detailed investigations in that field are required to gain a more holistic view on the issue of 2PA enhancement upon aggregation.

Bearing in mind that aggregated, insoluble nature of macromolecular materials provides possible new ways to enhance 2PA response, and that new characterization techniques had to be invented to gain the access to their 2PA properties, one might consider insoluble materials as a particular offshoot of NLO chromophores. To emphasize unique characteristics of such materials we have recently branded them as nonlinear optical pigments (NLO pigments) [43]. The term ‘pigment’ is intentionally borrowed from dyestuff nomenclature, since in itself it amalgamates the two hallmark properties, i.e., strong absorption of certain wavelengths of light as well as particulate physical form combined with the lack of solubility in solvents [50]. The added term ‘NLO’ extends the classic definition of pigment beyond linear optics to nonlinear absorption of light that occurs only at very high light intensities - with the 2PA being a prime example. More generally, NLO pigments might be considered complementary to the already well-established, broad class of NLO dyes, which encompasses various chemical objects soluble in solvents, e.g., organic compounds, organometallics, dendrimers, linear polymers and biomolecules [29,51,52,53,54].

**Scheme 1:**
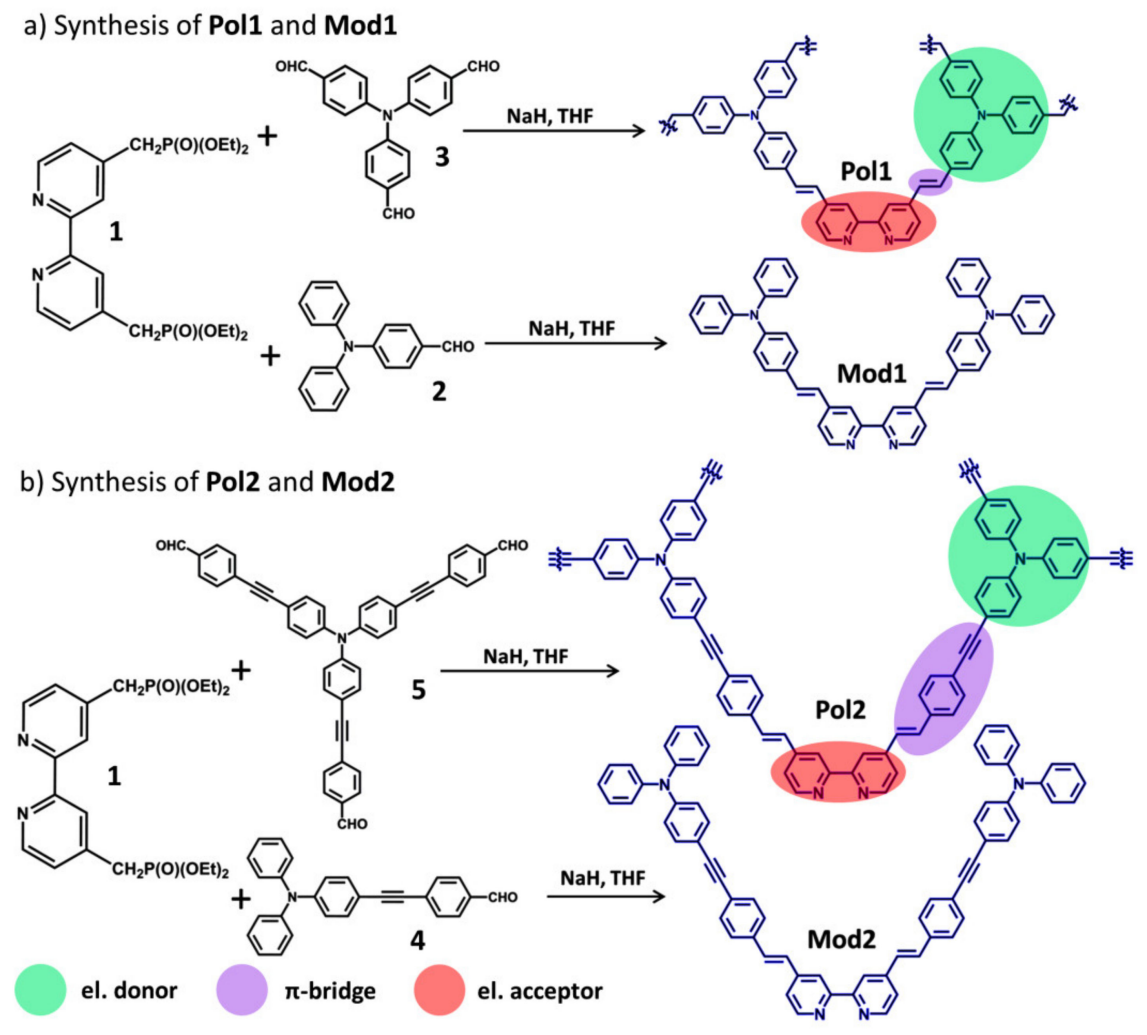
Synthetic schemes of (**a**) **Pol1** and **Mod1** (**b**) **Pol2** and **Mod2** demonstrating conditions and substrates used. Note that product compounds are stacked one above another to highlight structural similarities of model compounds versus respective polymers. With colored ovals are indicated structural fragments that are in the discussion referred to as electron donors, electron acceptors and π-bridges. For clarity of presentation these structural fragments have been highlighted only on one aromatic arm of **Pol1** and **Pol2** compounds.

The aim of this contribution is to investigate, for the first time, the spectrally-resolved 2PA properties of selected examples of crosslinked conjugated polymers, the emerging new members of NLO pigments class. To this end, we have synthesized new fluorescent crosslinked conjugated polymers based on triphenylamine and 2,2’-bipyridine aromatic cores (compounds **Pol1** and **Pol2**, see Scheme 1, vide infra) and characterized their 2PA properties in a broad wavelength range using both SSTPEF and ISTPEF techniques. In order to assess the impact of self-aggregation on the 2PA properties of these polymers, small-molecular models of corresponding polymers have been designed and prepared (see Scheme 1, **Mod1** and **Mod2)**, and their spectrally-resolved 2PA properties have been evaluated with the use of the classic TPEF technique in solution, as well as using SSTPEF technique on powdered samples. The evaluation of the effect of aggregated structure on the 2PA response of polymers and their small molecular models has been performed by the analysis of molar-mass normalized 2PA merit factors. Finally, in this report we demonstrate that *N*-coordination sites of 2,2’-bipyridine fragments of polymer **Pol1** can be postsynthetically coordinated by Eu^3+^ ions, thus introducing additional centers of luminescence. The obtained Eu^3+^-functionalized material, **Pol1-Eu** has been explored for possible applications in non-contact luminescence thermometry by investigation of its temperature-resolved luminescence properties under one-, two-, and three-photon excitation conditions.

## 2. Results and Discussion

### 2.1. Design and Synthesis of Crosslinked Polymers Pol1 and Pol2 and of Model Compounds Mod1 and Mod2

There are several requirements that served as our signposts in the selection of ideal candidate materials for spectrally-resolved studies of 2PA of crosslinked conjugated polymers. First, in order to ascertain the crosslinked character of polymer network, the target polymer material must possess a molecular unit capable of crosslinking. The second requirement is related to the emission properties of the target polymer. Since the determination of 2PA cross sections of NLO pigments employed in this work relies on the fluorescence-based techniques (SSTPEF and ISTPEF), the target material, to be of any use for such studies, must provide luminescence output that can be excited under two-photon conditions in a wide range of excitation wavelengths; in other words, the NLO pigment must also be an NLO luminophore (NLO phosphor). One more structural feature that should be ideally met is the presence of functional groups that will maximize the 2PA response. To this end, structural components of target polymers have been chosen to benefit from known design principles of NLO chromophores, such as donor-acceptor structure and π-conjugation [55,56].

As a result of screening studies we found that the above requirements are met by polymers containing electron-donating (triphenylamine) and electron-withdrawing (2,2’-bipyridine) structural fragments joined by vinylene (**Pol1**) or vinyl(4-ethynylphenyl) (**Pol2**) aromatic bridges (Scheme 1). As seen, these two polymers that differ in the length of π-conjugated aromatic arms were chosen. This choice was dictated by the need to verify whether the elongation of π-bridges in such systems influences their 2PA properties, as well as to probe the effect of an organic arm length on the steady state and time-resolved emission properties of the resulting polymers. 

An evaluation whether the self-aggregation of crosslinked conjugated polymers has a beneficial effect on 2PA properties is not a trivial matter. The fundamental obstacle is that resulting polymer structures are largely different in terms of the size of aromatic backbone from starting materials used for their synthesis. This feature differentiates polymers from CPs, as in the latter case the ligands generally preserve their discrete, molecular character when they are incorporated into the metal-organic chain via coordination bonds. Consequently, preserved chemical identity of ligand molecules allows one to make direct comparisons of 2PA properties between free ligands and corresponding CPs, whereas for polymers a different strategy must be devised. In order to circumvent this issue we designed new organic chromophores (Scheme 1, **Mod1**, 4,4′-bis-[(*E*)-2-(4-diphenylaminophenyl)ethenyl]-2,2′-bipyridine and **Mod2**, 4,4′-bis-[(*E*)-2-(4-diphenylaminophenyl-(4-ethynylphenylene))ethenyl]-2,2′-bipyridine) whose structures are intended to be small, molecular models representing the main structural motifs present in organic backbones of **Pol1** and **Pol2. Mod1** and **Mod2** are obtained as yellowish solids and are soluble in chlorinated solvents, allowing us to investigate their 2PA properties in solution using TPEF technique as well as in the solid state, using SSTPEF technique. Results obtained with the latter technique can be used for comparisons of 2PA responses of **Pol1** and **Pol2** with those of **Mod1** and **Mod2**.

From the synthetic standpoint, **Pol1** and **Pol2** were obtained by employing Horner–Wadsworth–Emmons reaction of triformyl derivatives of triphenylamine (tris(4-formylphenyl)amine (**3**) and tris-4,4’,4’’-(4-formylphenylethynyl)triphenylamine (**2**), respectively) with 4,4’-bis(diethoxyphosphorylmethyl)-2,2’-bipyridine (**1**). Owing to the presence of three formyl groups at 4, 4’ and 4’’ sites, the triphenylamine-based compounds served as three-connected crosslinking nodes, as required by delineated above design guidelines, whereas phosphonate diester acted as precursor for electron withdrawing π-bridge. The conjugated character of the final polymers is ensured by the presence of double bonds that are created during carbon-carbon coupling reaction. Small molecular models **Mod1** and **Mod2** were also obtained by employing Horner–Wadsworth–Emmons conditions, yet in those cases, **1** was reacted with mono-substituted formyl derivatives, i.e., 4-(diphenylamino)benzaldehyde (**4**) and 4-((4-(diphenylamino)-phenyl)ethynyl)benzaldehyde (**5**), respectively (Scheme 1).

Note that in all cases the Horner–Wadsworth–Emmons reaction was employed for formation of double bonds rather than the Wittig reaction. The immense advantage of the Horner–Wadsworth–Emmons olefination is that in the case of aromatic aldehydes it yields double bonds of (*E*)-disposition, by contrast to Wittig conditions which tend to afford double bonds of (*Z*) stereochemistry [57]. From the viewpoint of designing 2PA chromophores the former arrangement is much more desirable since it allows for larger overlap of sigma orbitals, thus offering higher conjugation of the π-electron system [56]. One more advantage of Horner–Wadsworth–Emmons protocol is that its phosphate byproducts are facile to remove with washing and Soxhlet extraction. This is in contrast to bulky triphenylphosphine-based compounds, typical for Wittig conditions. 

The obtained polymers **Pol1** and **Pol2** were amorphous in nature, as judged from continuous halo present in their powder X-ray diffraction patterns (Figure S1 in the Supplementary Information). Elemental analyses were in fair agreement with their proposed compositions. Nevertheless, one notes that experimentally determined percentage contents of C and N in dried samples of **Pol1** and **Pol2** are for both elements smaller (particular deviation is observed for the carbon content) than those calculated for idealized stoichiometries, suggesting that not all branches of triphenylamine building blocks are connected with corresponding 2,2’-bipyridine fragments and vice versa. It is therefore reasonable to assume that the part of organic arms does not participate in the formation of the polymer network, but serves as host for oxygen atoms of unreacted and/or partially transformed functional groups, thus decreasing the overall content of C and N elements.

### 2.2. Linear and Nonlinear Optical Properties of Model Compounds Mod1 and Mod2 in Chloroform Solution – TPEF Study

**Figure 1:**
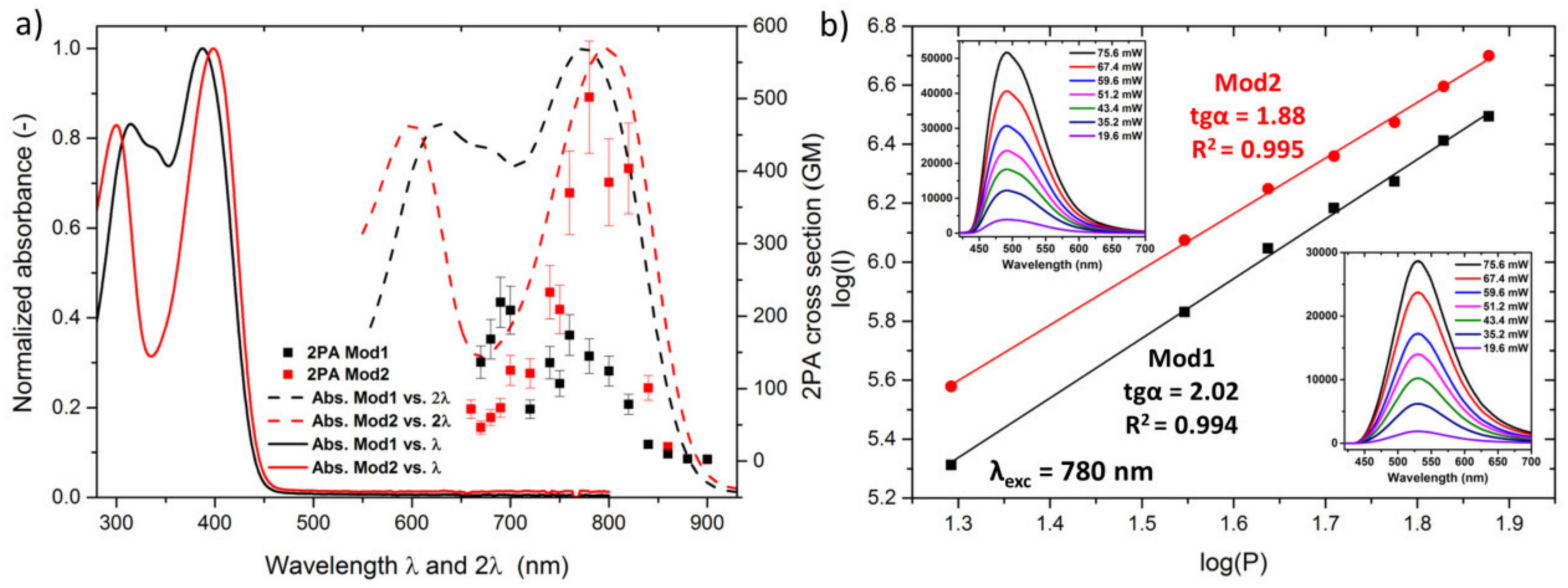
(**a**) Overlay of normalized one-photon absorption spectra (chloroform solution) plotted versus λ (full lines) and 2λ (dashed lines) with 2PA spectra of **Mod1** and **Mod2** measured using two-photon excited fluorescence (TPEF) technique. (**b**) Log−log plots of integral intensities of **Mod1** and **Mod2** emissions excited at 780 nm in the function of applied laser power. Corresponding emission spectra of **Mod1** and **Mod2** are provided as insets in lower right and upper left part of the plot, respectively.

Optical properties of molecular models **Mod1** and **Mod2**, measured in solution state (4.6·10^−6^ M in chloroform), will be discussed first. 2PA cross section spectra of **Mod1** and **Mod2** were determined using the TPEF technique in the range from 660 nm to 900 nm, using Rhodamine 6G in methanol (c = 8.0·10^−6^ M) as a 2PA reference [58]. As seen in Figure 1a, **Mod1** reveals two distinct 2PA cross section (σ_2_) maxima, one at 690 nm (219 GM) and the second one at 760 nm (173 GM). In the case of **Mod2** there is one prominent 2PA band, with a maximum of 502 GM at 780 nm. Note that **Mod2** features over twice higher σ_2_ value at maximum than **Mod1** does. This result is in line with our expectations, as the elongation of the π-conjugation path (here achieved by the introduction of 4-ethynylphenylene unit) is an effective approach to increase 2PA cross section values of NLO chromophores, since greater separation between charges significantly increases the hyperpolarizability of the system [55]. The advantageous effect of π-chromophore extension on 2PA performance is also reflected in so-called molar-mass normalized 2PA cross sections (σ_2_/M), which by far are the most widely used merit factors to assess the relative strength of nonlinear absorption processes [59]. Indeed, if maximal 2PA cross section values are divided by respective molar masses of chromophores, σ_2_/M values of 0.34 GM·mol·g^−1^ and 0.56 GM·mol·g^−1^ for **Mod1** and **Mod2** are obtained, respectively. Worth highlighting is that good 2PA properties of **Mod1** and **Mod2** are accompanied by high quantum yields (QY) of fluorescence (0.54 and 0.79, respectively), as checked by absolute measurements of QY in integration sphere using one-photon 377 nm excitation (Table 1, vide infra).

Figure 1a also presents normalized one-photon absorption spectra of **Mod1** and **Mod2** plotted versus λ (full lines) and 2λ (dashed lines), both measured as diluted chloroform solutions. It is apparent that the observed maxima of 2PA spectra of **Mod1** and **Mod2** correspond to their linear optical absorption maxima plotted against twice the wavelength. This behavior is expected for molecules of acentric structure, since for such systems one-photon-allowed transitions are also two-photon-allowed. For this reason, molecules **Mod1** and **Mod2** are drawn in Scheme 1 as noncentrosymmetric *cis* conformers (i.e., for which dihedral angle between adjacent N-C-C-N atoms is equal to 0 deg.), yet it must be emphasized that the single bond joining pyridyl rings is rotationally labile, giving rise to a number of different conformations, including centrosymmetric *trans* conformer. Indeed, DFT studies on 2,2’-bipyridine derivatives structurally related to **Mod1** and **Mod2** show that *trans* geometry (dihedral N-C-C-N angle of 180 deg.) is energetically more stable relative to *cis* one by 4 kcal/mol [60]. This rather low energy difference suggests that populations of different conformers might be present in solutions of 2,2’-bipyridine-based NLO chromophores, such as **Mod1** and **Mod2**. It follows that conformers deviating from the perfectly centrosymmetric *trans* geometry are likely to contribute to the observed 2PA spectra.

The assumed 2PA character of observed emissions has been verified by power-dependent fluorescence intensity measurements. In Figure 1b are presented logarithmic plots of the fluorescence intensity dependence on the input laser power for **Mod1** and **Mod2** chloroform solution, both measured upon excitation at 780 nm. Slopes of these plots are both close to the value of 2, confirming the two-photon origin of these emissions.

### 2.3. Linear and Nonlinear Optical Properties of Model Compounds Mod1 and Mod2 in Solid State and of Crosslinked Polymers Pol1 and Pol2 – ISTPEF and SSTPEF Studies

2PA measurements presented in preceding section were performed for diluted chloroform solutions of model chromophores **Mod1** and **Mod2,** hence the obtained 2PA spectra provide a measure of nonlinear absorption of individual dye molecules, i.e., for which the influence of aggregation effects on 2PA is largely suppressed. In this section we proceed to the characterization of 1PA and 2PA properties of compounds that are available as solid state phases, hence their 2PA properties are expected to be influenced by aggregation effects. 

**Figure 2:**
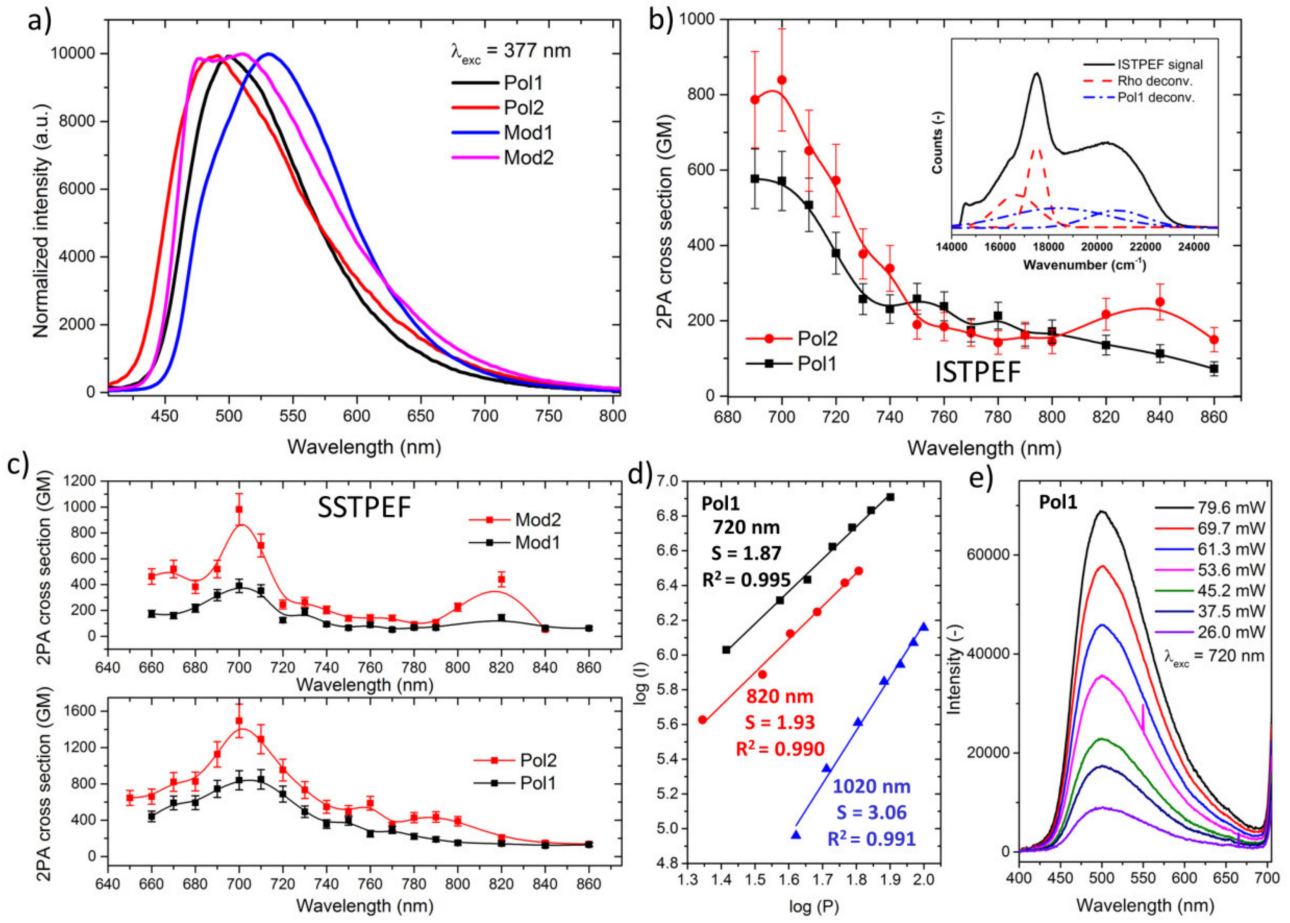
(**a**) Overlay of normalized emission spectra of **Mod1**, **Mod2**, **Pol1**, **Pol2** (λ_exc._ = 377 nm). (**b**) Overlay of 2PA spectra for **Mod1** and **Mod2** measured with the use of internal standard TPEF (ISTPEF) technique. Inset presents an example of experimental ISTPEF spectrum (drawn as black line) obtained upon 820 nm laser excitation. Contributing emissions of **Pol1** and Rhodamine B are drawn as Gaussian deconvolution functions in blue and red dashed lines, respectively. Note that deconvolution procedure was performed for emission spectra plotted in wavenumber scale. (**c**) Upper panel: overlay of 2PA spectra for **Mod1** and **Mod2** measured with the use of SSTPEF technique. Lower panel: overlay of 2PA spectra for **Pol1** and **Pol2** measured with the use of SSTPEF technique. (**d**) Log−log plots of integral intensities of **Pol1** emissions excited at 720 nm, 820 nm and 1020 nm in the function of applied excitation laser power. Slopes indicate 2PA as the origin of emissions at 720 nm and 820 nm and 3PA at 1020 nm. (**e**) Emission spectra of **Pol1** measured in the function of the applied excitation laser power (λ_exc._ = 720 nm).

In Figure 2a are presented solid state fluorescence spectra of **Mod1**, **Mod2**, **Pol1**, and **Pol2** obtained upon linear excitation at 377 nm. It appears that maxima of fluorescence for polymers **Pol1** and **Pol2** are shifted to shorter wavelengths relative to **Mod1** and **Mod2** (note that in the latter case the solid state emission spectrum reveals two distinct bands). The blueshift of emission band is bigger for **Mod1-Pol1** pair (Δλ = 39 nm) than for **Mod2-Pol2** pair (Δλ = 33 nm). At present it is not clear whether the observed blueshift of emission maxima for polymer samples is due to extended character of the aromatic system relative to model compounds, or rather stems from different aggregation modes of aromatic arms in these two classes of solids. Additionally, when performing steady state fluorescence measurements we observed that the emission intensities of crosslinked polymers are much weaker compared to corresponding model compounds in the solid state form. In numbers, absolute fluorescence QYs were found equal to 0.32, 0.16, 0.11 and 0.07 for **Mod1**, **Mod2**, **Pol1** and **Pol2**, as measured under linear excitation (377 nm). These values quantitatively demonstrate that polymer samples feature 2-3 times lower fluorescence QYs than corresponding model compounds. It is also evident that the pair of compounds bearing vinyl(4-ethynylphenyl) π-bridge (**Mod2** and **Pol2**) offers lower QY values than the corresponding **Mod1** and **Pol1** analogues with vinyl π-bridge. This comparison suggests that vinyl(4-ethynylphenyl) fragment introduces some radiationless relaxation pathways that bring about the decrease of QY. 

The next step was the characterization of 2PA properties with the use of ISTPEF and SSTPEF techniques. ISTPEF measurements of **Pol1** and **Pol2** have been performed by excitation (690 nm–860 nm with 20 nm step, 1 kHz laser pulses) of polymers dispersed in methanolic solution of Rhodamine B. Simultaneous collection of the emission spectrum of crosslinked polymers and that of Rhodamine B obviates the issue of different scattering factors [43], but also necessitates deconvolution of the emission signals at each excitation wavelength employed. In Figure 2b (inset) is provided representative ISTPEF signal of **Pol1** obtained at 820 nm excitation, plotted along with Gaussian deconvoluting curves. Calculations of 2PA cross sections have been performed per C_39_H_27_N_4_ and C_63_H_39_N_4_ formulas, for **Pol1** and **Pol2**, respectively. These formulas are representations of the smallest repeating units of these polymers and comprise one three-connected triphenylamine-based fragment of the polymer connected with one and half of the 2,2’-bipyridine fragment along with corresponding π-bridge. 2PA spectra for **Pol1** and **Pol2** measured with ISTPEF demonstrate that for both compounds their maximal cross section values (586 GM and 839 GM, respectively) are located at ca. 700 nm, while in the case of **Pol2** one can also distinguish an additional maximum at 840 nm (283 GM). Note that spectra presented in Figure 2b appear to be ‘truncated’ on their short-wavelength edges. This is because we could not measure 2PA values for excitation wavelengths shorter than 690 nm since at those wavelengths the tail of laser radiation starts to overlap with the fluorescence signal of Rhodamine B, and it is not possible to effectively remove its contribution from the collected signal. Solid samples of **Mod1** and **Mod2** have not been investigated with the use of ISTPEF technique due to their partial solubility in methanol.

SSTPEF measurements have been performed for **Mod1**, **Mod2**, **Pol1**, and **Pol2** using powdered sample of bis(4-diphenylamino)stilbene (BDPAS) as a 2PA standard. By contrast to ISTPEF results, 2PA spectra obtained with SSTPEF provide information on 2PA cross sections for wavelengths shorter than 690 nm (650–860 nm measurement range), as displayed in Figure 2c. Owing to broader measurement range, based on these results it can be concluded that maximum 2PA response for all compounds is indeed located at around 700 nm, while for **Mod2** and **Pol2** one can also find the additional maxima at 820 nm and 800 nm, respectively. Calculated 2PA cross sections of polymers are higher than those of corresponding model compounds. For example, maximal σ_2_ values for **Pol1** and **Mod1** are equal to 850 GM and 390 GM, respectively, while for **Pol2** and **Mod2** maximal σ_2_ values are equal to 1494 GM and 982 GM, respectively. From this comparison it is also clear that π-extended analogues (**Pol2** and **Mod2**) offer higher 2PA cross sections relative to **Pol1** and **Mod1**, confirming what was observed in ISTPEF results for polymer samples (Figure 2b).

In a similar manner as we did in the analysis of TPEF results of **Mod1** and **Mod2** (Section 2.1), we have performed a normalization procedure of the 2PA cross sections of investigated polymers and model compounds by calculating molar mass-normalized ‘σ_2_/M’ merit factors (Table 1) [59,61]. A comparison of these values reveals that **Pol1** and **Pol2** feature σ_2_/M values of 1.42 and 1.67, whereas for model compounds **Mod1 and Mod2** those parameters take values of 0.56 and 1.10, respectively. The particular enhancement is observed for **Pol1**-**Mod1** pair, with nearly three-fold difference in σ_2_/M values. In the case of **Pol2** and **Mod2** the σ_2_/M value for the former one is higher by ca. 50%. We take the above results as a proof that conjugated polymers **Pol1** and **Pol2** indeed feature enhanced 2PA response in comparison to corresponding model compounds in solid state form. 

This is the first report in which 2PA cross sections of solid state materials are independently investigated using two different measurement techniques, i.e., SSTPEF and ISTPEF. Therefore, a closer examination of similarities and differences in obtained 2PA spectra may provide useful information, important from the methodological point of view. Indeed, the comparison of σ_2_ values tabulated in Table 1 shows that 2PA cross sections determined with the use of SSTPEF technique tend to be higher than corresponding ones from ISTPEF. For example, at global maxima the 2PA cross section values are equal to 850 GM vs. 576 GM for **Pol1** and 1494 GM vs. 839 GM for **Pol2**. Moreover, when looking at 2PA spectra it is clear that, while the positions of maxima are located at the same wavelengths, the relative ratio between local maxima is slightly different. These differences might seem surprising at first, but they can be easily explained by consideration of experimental conditions that are employed in each of these techniques. In the case of ISTPEF technique the 2PA cross section is calculated versus a two-photon standard dye, whose dispersion of 2PA cross sections was precisely determined in solution. In the case of SSTPEF technique, the 2PA cross section is calculated against solid state sample of a reference dye, here bis(4-diphenylamino)stilbene. The use of solid state reference has an underlying assumption that the 2PA cross sections of the standard dye, which were determined in solution, are the same for pure, solid state sample. While the 2PA cross sections of references that are used for measurements are values linearly extrapolated to the pure substance, such an extrapolation does not account for possible effects that may change the actual 2PA of the reference dye in the solid state. Therefore, some differences between 2PA cross sections obtained from SSTPEF and ISTPEF can be expected. If rigorous comparison of 2PA responses of different materials is required, then the most telling assessment can be made if all samples are measured according to the same protocol, if possible. In the case of studies here compounds, only SSTPEF technique was applicable to all compounds, whereas ISTPEF could be employed to **Pol1** and **Pol2** but not **Mod1** and **Mod2**, owing to the partial solubility of the latter two compounds in methanol.

Power-dependent fluorescence measurements on solid samples of polymers helped in confirming the nature of nonlinear photon absorption processes at two wavelength regions where local emission maxima were found. Log-log plots of the integral emission intensities at these wavelengths in the function of incident beam intensity for **Pol1** are shown in Figure 2d. Linear fittings of those plots prove the anticipated two-photon character of emissions excited at 720 nm and 820 nm. In Figure 2e are presented emission spectra of **Pol1** collected during power-dependent experiment at 720 nm. Additionally, we have performed power-dependent fluorescence measurements on **Pol1** at wavelengths longer than the upper limit of our 2PA spectra (860 nm). For example, upon excitation of **Pol1** with 1020 nm beam we have observed a fluorescence signal, which revealed a cubic dependence on the intensity of input laser power (see Figure 2d, and Figure S2 in the Supplementary Information for experimental spectra). An analogous set of power-dependent measurements at 720 nm, 820 nm, and 1020 nm has been conducted on **Pol2**. It turns out that the origin of emissions at each of the investigated wavelengths (Figure S3 in the Supplementary Information) mirrors that of **Pol1**. Only in the case of **Pol2** we have observed a weak fluorescence signal, overlapped with third-harmonic of exciting radiation (λ_THG_ = 450 nm, Figure S4b in the Supplementary Information), when the sample was irradiated with 1350 nm beam (for **Pol1** only the THG signal was present, Figure S4a in the Supplementary Information). Intensity of this fluorescence signal was much too weak to perform reliable power-dependent experiment. Nevertheless, considering the spectral range at which the NLO excitation takes place (approximately twice the wavelength where 2PA occurs) it may be supposed that at 1350 nm four-photon absorption (4PA) is a likely scenario.

Next, we proceeded to analysis of other relevant parameters that describe 2PA and related properties of investigated solids. From the viewpoint of applications, a particularly relevant quantity is the two-photon brightness (σ_2_φ, sometimes also referred to as active or effective two-photon action cross section), which expresses the strength of luminescence that can be obtained upon two-photon excitation conditions. Indeed, despite high values of 2PA cross-sections and molar-mass normalized 2PA merit factors for **Pol1** and **Pol2**, the σ_2_φ values for these polymers are noticeably smaller than for the low-molecular model compounds **Mod1** and **Mod2** in the solid state (Table 1). It is evident that the QY component is responsible for relatively low σ_2_φ values. Indeed, QYs determined for solid state samples **Mod1** and **Mod2** are lower compared to those of **Mod1** and **Mod2** obtained in diluted chloroform solutions (1.6-fold, and 1.9-fold decrease, respectively). Additionally, QYs for **Pol1** and **Pol2** are the lowest among all samples investigated and are equal to 0.11 and 0.07, respectively (Table 1). These data suggest that the investigated systems suffer from the aggregation-caused quenching (ACQ) phenomenon [62]. ACQ is occasionally encountered in compounds that form short intermolecular interactions between molecules, e.g., π–π stacking between aromatic fragments. For some unfavorable chromophore arrangements non-radiative relaxation pathways are introduced, resulting in quenching of excited states. To verify the deleterious effect of aggregated structure on the excited state dynamics, the fluorescence decay lifetime data have been collected for solutions of models and solid state samples using time-correlated single photon counting (TCSPC) at room temperature (see Figure S5 in the Supplementary Information for experimental decay curves). For **Mod1** and **Mod2** in diluted CHCl_3_ solutions one notes clean single-exponential luminescence decay curves with time constants of 1.98 ns and 2.04 ns, whereas for **Mod1** and **Mod2** in solid state form, decay curves require the use of three-exponential models, with average lifetimes of 1.92 ns and 1.70 ns, respectively. Decay data for **Pol1** and **Pol2** also had to be fitted using three-exponential models, yielding average lifetimes as low as 0.79 ns and 0.77 ns. Thus, significantly shortened fluorescence lifetimes of **Pol1** and **Pol2** compared to their respective solid state molecular models suggest that, indeed, the ACQ phenomenon is operative here. 

A complete picture of relative strength of 2PA-mediated fluorescence properties of **Pol1** and **Pol2** can be obtained if one performs the normalization of two-photon brightness values by the molar mass of molecule/repeating unit to get molar mass-normalized two-photon brightness value (σ_2_φ/M). This procedure aims at determining whether the intensity of 2PA-excited fluorescence (not the 2PA cross section itself) is improved or not. Here, comparison will be made relative to model compounds. The calculated values of molar mass-normalized two-photon brightness values (σ_2_φ/M) for **Mod1**, **Mod2**, **Pol1** and **Pol2** are tabulated in Table 1. If one compares σ_2_φ/M values calculated using SSTPEF-based 2PA cross sections it is apparent that the polymer samples feature lower values (0.16 and 0.12 for **Pol1** and **Pol2**, respectively) than their model compounds (0.18 for both **Mod1** and **Mod2**). Accordingly, despite improved intrinsic 2PA response, the net effect on strength 2PA-induced emission is disadvantageous, as the effective intensity of two-photon excited emission for **Pol1** and **Pol2** is slightly lower relative to model compounds **Mod1** and **Mod2**. 

The take-home message that can be derived from the above results is that, apart from efforts directed on maximalization of the intrinsic 2PA cross section values, it is also crucial to preserve as high as possible quantum fluorescence efficiency in order to obtain maximal values of the two-photon brightness (σ_2_φ). One presumable solution to that issue may be to introduce into the polymer structure certain organic fragments, e.g., tetraphenylethene core, that are likely to reveal aggregation-induced emission (AIE), an effect opposite to ACQ [63].

### 2.4. Postfunctionalization With Eu^3+^ Ions: Evaluation of One-, Two-, and Three-Photon Temperature Probes

In preceding sections, we have shown that crosslinked conjugated polymers **Pol1** and **Pol2** are good two-photon absorbers, yet their up-converted fluorescence is of rather fair intensity due to deleterious ACQ phenomenon. Nevertheless, for these compounds we have been able to collect up-converted emissions not only those due to 2PA excitation, but also for longer exciting wavelengths, e.g., the 3PA-mediated fluorescence was also detected with good signal-to-noise ratio when exciting at 1020 nm. This opens up the question whether excitation in near-infrared range can be leveraged e.g., for construction of NLO fluorescent probes. Some indication on how the NLO-induced luminescence can be harnessed for external stimuli sensing has been presented in our previous contribution, in which we demonstrated that the other representatives of NLO pigments—CPs built from lanthanide ions (Gd^3+^ as matrix and Eu^3+^, Tb^3+^ ions as dopants), 1,3,5-benzenetricarboxylic acid and phenantroline—can serve as NLO luminescent thermometers [48]. Specifically, ligands building up CPs were excited using 800 nm femtosecond laser pulses. Energy harvested in this way yielded temperature-dependent emissions from lanthanide centers. Thus, by employing the 3PA of CP, the NIR-to-VIS non-contact ratiometric luminescent sensing of temperature was made possible in materials which previously were excited exclusively in the linear optical regime, e.g., with the use of ultraviolet [64].

Based on this experience we conducted a preliminary exploration whether a similar NLO functionality can be imparted onto presented here crosslinked conjugated polymers. If one looks at emission spectra of **Pol1** and **Pol2** it is clear that their fluorescence envelopes do not contain well-resolved bands, hence it is practically impossible to select two fragments of emission spectrum whose thermal quenching kinetics would be different. For this reason, neat polymers **Pol1** and **Pol2** are of little use for ratiometric luminescent thermometry. Accordingly, in order to introduce an additional source of emission of disparate thermal quenching character, we added a lanthanide component to our material. Specifically, we conducted postfunctionalization of polymer **Pol1** by refluxing it with europium complex (europium tris(acetylacetonate)) in methanol, yielding partially complexed polymer (**Pol1-Eu**). Postfunctionalization reaction was possible owing to the presence of 2,2’-bipyridine fragments in the polymer structure, whose nitrogen atoms served as coordination sites to which Eu^3+^ ions are anchored. It should be noted that despite a prolonged reaction time (24 h) and large, ten-fold excess of europium source the overall content of Eu^3+^ in **Pol1-Eu** was found to be as low as 1.23%, (mass content, as determined from inductively coupled plasma optical emission spectrometry (ICP-OES)). This percentage content indicates that Eu^3+^ centers coordinate only to approx. 3.1% of available 2,2’-bipyridine fragments, which may be due to hindered access of these ions to inner parts of the polymer. Attachment of Eu^3+^ is also reflected in luminescence properties of this material. Figure S6 in the Supplementary Information displays the spectrum of **Pol1-Eu**, obtained upon 377 nm excitation. The spectrum contains two emissions of disparate nature, as planned: at room temperature the broad envelope of the organic backbone emission with maximum at 500 nm as well as a set of Eu^3+^ bands originating from radiative decay of its ^5^D_0_ state: 590 nm (^5^D_0_→^7^F_1_), 615 nm (^5^D_0_→^7^F_2_), 650 nm (^5^D_0_→^7^F_3_), and 698 nm (^5^D_0_→^7^F_4_). Note that emission from ^5^D_0_→^7^F_0_ transition, expected to be at ca. 576 nm is not discernible in our dataset, but this is not surprising given that this band is generally weak due to its spin and electric dipole forbidden character. The emission from ^5^D_0_→^7^F_2_ transition has the highest intensity among all Eu^3+^ bands, indicating that chromophoric environments of coordinated Eu^3+^ ions are acentric [65]. Functionalization with Eu^3+^ ions did not affect amorphous nature of the resulting polymer (Figure S7 in the Supplementary Information).

**Figure 3:**
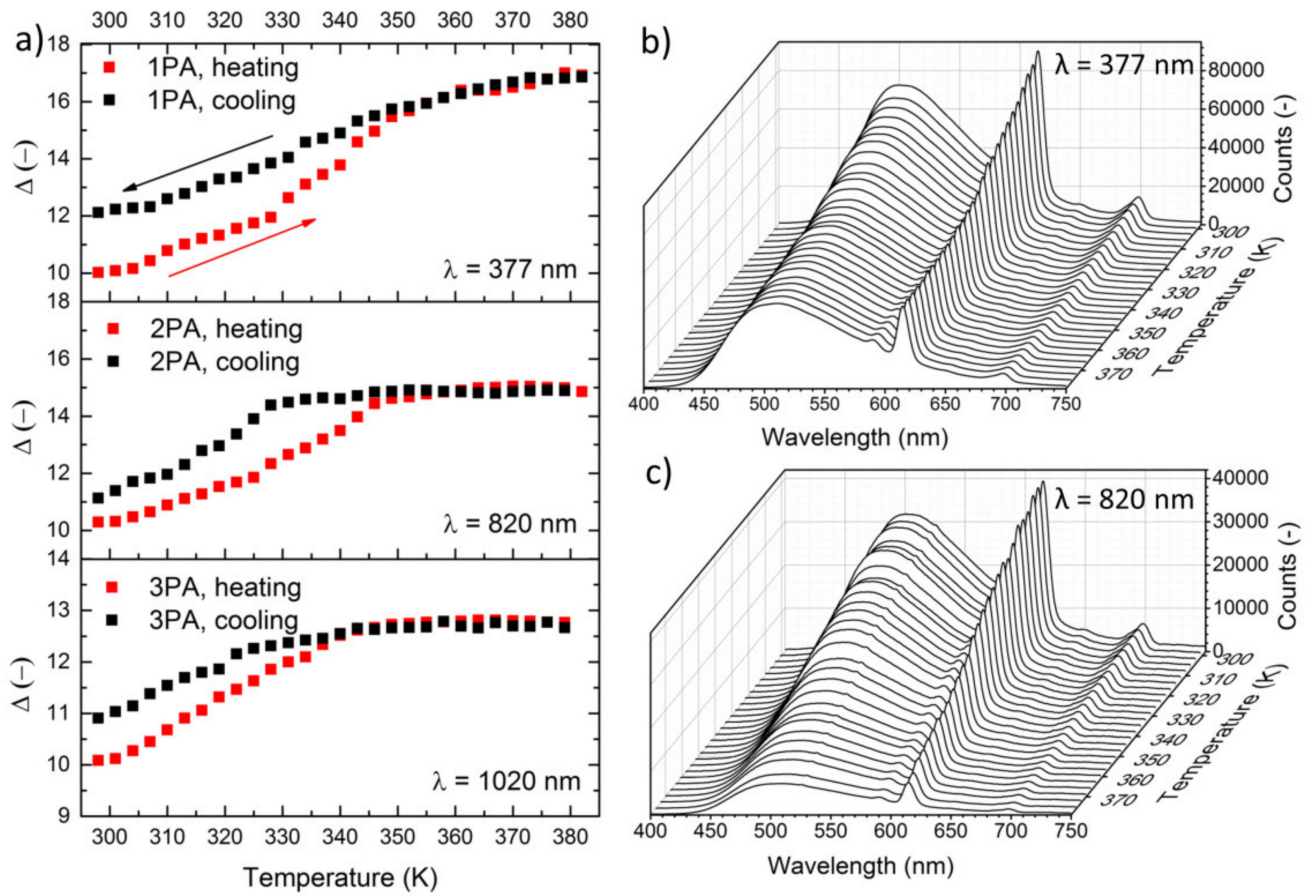
(**a**) Plots of one-photon (upper panel), two-photon (middle panel) and three-photon (lower panel) thermometric parameters plotted in the function of temperature for **Pol1-Eu**. Data for heating and cooling runs are presented as red and black squares, respectively. (**b**) Experimental spectra of **Pol1-Eu** obtained during heating run of temperature-resolved experiment under linear excitation conditions (λ_exc._ = 377 nm). (**c**) Experimental spectra of **Pol1-Eu** obtained during heating run of the temperature-resolved experiment under two-photon excitation conditions (λ_exc._ = 820 nm).

Next, we investigated the emission behavior of **Pol1-Eu** in the function of temperature, by employing different excitation modes: 1PA (377 nm), 2PA (820 nm), and 3PA (1020 nm). In order to quantitatively express changes in relative intensities in the collected emission spectra, for each spectrum we calculated thermometric parameters, Δ, defined here as the ratio of integral area of S_1_→S_0_ emission of the organic component (integrated in 420–570 nm range) to ^5^D_0_→^7^F_2_ (Eu^3+^) *f*-*f* transition component. The latter emission was integrated in 601–631 nm range, after subtraction of the background emission. Corresponding plots of one-photon, two-photon, and three-photon thermometric parameters plotted in the function of temperature (298–382 K) for **Pol1-Eu** are displayed in Figure 3a. Experimental spectra of **Pol1-Eu** obtained upon heating runs of the temperature-resolved experiments under one- and two-photon excitation conditions are provided in Figure 3b,c.

Excitation of **Pol1-Eu** with 377 nm beam in the 298–382 K range (with 3K step) has shown that, as temperature rises, the emission of Eu^3+^ is thermally quenched to a bigger extent than that from the polymer component (Figure 3a). Indeed, in the investigated temperature range, in the heating run the Δ values rise from 10 to nearly 17, which corresponds to ca. 40% drop of Eu^3+^ intensity versus the organic reference. In the heating run one can see two inflection points, one at ca. 330K, and the second one at 360K. Interestingly, upon cooling run Δ values follow those obtained upon heating run down to 350K; below this point Δ values start to deviate more and more from the corresponding ones measured for the heating run. 

Plot of Δ vs. temperature for data obtained from two-photon experiment (820 nm) on **Pol1-Eu** is presented in the middle panel of Figure 3a. Similarly to one-photon temperature-resolved experiment, the temperature rise results in the increase of Δ value, but in this case this parameter reaches plateau at ca. 350 K that continues up to 379 K. This behavior is a bit different compared to 1PA excitation and suggests that the excitation mode (specifically, the laser radiation) itself affects in some way the emission properties of the material. Indeed, the visual inspection of the **Pol1-Eu** after thermometric experiment at 820 nm has shown brownish spots in the irradiation area, indicating that elevated temperature, combined with prolonged exposure to laser radiation, triggers a photochemical decomposition of this material. 

Results of three-photon thermometric experiment are displayed in Figure 3a, lower panel. The shape of Δ = f(T) plot qualitatively reproduces what was observed for the experiment conducted at two-photon excitation conditions. Note, however, that the calculated values of thermometric parameters rise more slowly in the function of temperature: for example, the Δ value at 340 K is equal to 12.8 (excitation at 1020 nm, 3PA) compared to 14.3 (excitation at 820 nm, 2PA). In this case, the photochemical decay of the sample was also evident, and appeared to be more intense. This might be due to relatively higher fluence needed to obtain sufficiently strong emission signal. 

Taken together, the luminescence of **Pol1-Eu** produced by employing 1PA, 2PA, and 3PA-mediated excitation modes is responsive to temperature changes. Nevertheless, the observed temperature characteristics of Δ values are in each case different for heating and cooling runs. It follows that the investigated material does not fulfill a fundamental condition of signal reversibility, required for any luminescent thermometer [66]. The origin of the lack of signal reversibility is not clear at present, but the fact that the registered Δ = f(T) plot could not be described with relatively simple phenomenological relationships suggests that many different relaxation pathways might be involved into the thermal decay of the luminescence of both emission centres: polymeric backbone and Eu^3+^ ions. Furthermore, some temperature-induced changes in the polymer structure are a possible explanation for the lack of signal reversibility. Additionally, strongly prohibitive is some sensitivity of **Pol1-Eu** material to the exposure to laser radiation emerging at temperatures higher than ca. 330–340 K. Taken together, this preliminary investigation of thermometric properties of **Pol1-Eu** demonstrates that it is feasible to obtain temperature-dependent two- and three-photon excited luminescence for this material, but crosslinked conjugated polymers devoid of the above shortfalls should be searched. 

## 3. Materials and Methods 

### 3.1. General Methods

Nuclear magnetic resonance (NMR) spectra were recorded on a Jeol JNM-ECZ 400S Research FT NMR spectrometer (JEOL Ltd., Tokyo, Japan) operating at 400 MHz (for ^1^H). Mass spectra were recorded using a Waters LCT Premier XE mass spectrometer (Milford, MA, USA). Electrospray ionization in positive ion mode was employed. Elemental analysis was performed using a CE Instruments CHNS 1110 elemental analyzer (Milano, Italy). Absorption spectra in the UV–Vis region were acquired in 1 cm quartz cuvettes using a Jasco V-670 spectrophotometer (Easton, MD, USA). Powder X-ray diffraction (PXRD) patterns of the conjugated crosslinked polymers were measured on a PANanalytical X’Pert diffractometer (Almelo, The Netherlands) equipped with a Cu-Kα radiation source (λ=1.54182 Å). Inductively coupled plasma optical emission spectrometry (ICP-OES) analysis of digested samples of europium-chelated crosslinked conjugated polymer was conducted in order to determine the content of the Eu element. This was performed using a Thermo Fisher Scientific iCAP 7400 spectrometer (Waltham, MA, USA) on **Pol1-Eu** sample digested in nitric acid-perhydrol mixture (10:1 by volume). Mineralization was performed under microwave heating conditions (MAGNUM II *ERTEC* MV 02-02 reactor, Wrocław, Poland)

### 3.2. Materials

Starting materials were of reagent grade purity and were obtained from commercial sources (Sigma-Aldrich, St. Louis, MO, USA and Avantor Chemicals, Gliwice, Poland) and used without further purification. THF was kept over 3Å Aldrich molecular sieves. Syntheses of compounds **1**–**5** and their precursors are provided in the Supplementary Information. 

Synthesis of 4,4′-bis-[(*E*)-2-(4-diphenylaminophenyl)ethenyl]-2,2′-bipyridine (**Mod1**). In the flask was placed 4,4’-bis(diethoxyphosphorylmethyl)-2,2’-bipyridine (**1**, 0.724 g, 1.59 mmol), 4-(diphenylamino)benzaldehyde (**2**, 1.300 g, 4.76 mmol), and dried THF (30 cm^3^). After dissolution of those reagents, NaH was added under nitrogen cushion (0.172 g of 60% NaH in mineral oil, 4.29 mmol of pure compound). Reaction mixture was stirred at room temperature overnight under dry nitrogen atmosphere, and then refluxed for 4 h. After cooling down, the reaction mixture was poured into water (100 cm^3^). Yellow precipitate was filtered out and dried on air. Crude product was extracted three times with boiling methanol (3 × 50 cm^3^). Light-orange methanolic extracts were discarded; the remaining yellow solid was subjected to column chromatography on silica gel chloroform/hexane = 5:1 (*v*/*v*) as an eluent, to afford yellowish solid in 64% yield (0.705 g). ^1^H NMR (400 MHz, CDCl_3_, 300 K) 8.64 (2 H, d, *J* = 5.2 Hz), 8.51 (2 H, s), 7.43 (4 H, d, *J* = 8.7 Hz), 7.41 (2 H, d, *J* = 16.3 Hz), 7.37 (2 H, dd, *J* = 5.2, 1.6 Hz), 7.29 (8 H, t, *J* = 8.0 Hz), 7.13 (8 H, dd, *J* = 8.6, 1.1 Hz), 7.09 (8 H, m), 7.01 (2 H, d, *J* = 16.3 Hz). HRMS ([M+H^+^], ES+) calc. 695.3165 for C_50_H_38_N_4,_ obs. 695.3174. Anal. Calcd (%) for C_50_H_38_N_4_: C, 86.42; H, 5.51; N, 8.06. Found: C, 86.11; H, 5.34; N, 7.92.

Synthesis of 4,4′-bis-[(*E*)-2-(4-diphenylaminophenyl-(4-ethynylphenylene))ethenyl]- 2,2′-bipyridine (**Mod2**). In the flask was placed 4,4’-bis(diethoxyphosphorylmethyl)-2,2’-bipyridine (**1**, 0.680 g, 1.49 mmol), 4-((4-(diphenylamino)-phenyl)ethynyl)benzaldehyde (**4**, 1.388 g, 3.72 mmol), and dried THF (30 cm^3^). After dissolution of those reagents, NaH was added under nitrogen cushion (0.161 g of 60% NaH in mineral oil, 4.02 mmol of pure compound). Reaction mixture was stirred at room temperature overnight under dry nitrogen atmosphere, and then refluxed under flowing nitrogen for 6 h. After cooling down the reaction mixture was poured into water (60 cm^3^). Yellow precipitate was filtered out and dried. Crude product was extracted three times with boiling methanol (3 × 50 cm^3^). Light-orange methanolic extracts were discarded; the remaining yellow solid was subjected to column chromatography on silica gel chloroform/hexane = 4:1 (*v*/*v*) as an eluent, to afford yellowish solid in 48% yield (0.639 g). ^1^H NMR (400 MHz, CDCl_3_, 300 K) 8.69 (2 H, d, *J* = 5.1 Hz), 8.57 (2 H, s), 7.54 (8 H, s), 7.46 (2 H, d, *J* = 16.3 Hz), 7.39 (4 H, d, *J* = 8.8 Hz), 7.41 (2 H, dd, *J* = 4.4, 1.2 Hz), 7.28 (8 H, t, *J* = 8.0 Hz), 7.16 (2 H, d, *J* = 16.3 Hz), 7.12 (8 H, dd, *J* = 8.6, 1.1 Hz), 7.07 (4 H, tt, *J* = 7.3, 1.1 Hz), 7.02 (4 H, d, *J* = 8.8Hz). HRMS ([M+H^+^], ES+) calc. 895.3809, obs. 895.3801. Anal. Calcd (%) for C_66_H_46_N_4_: C, 88.56; H, 5.18; N, 6.26. Found: C, 88.40; H, 5.01; N, 6.15.

Synthesis of crosslinked conjugated polymer **Pol1**. In the flask was placed 4,4’-bis(diethoxyphosphorylmethyl)-2,2’-bipyridine (**1**, 0.750 g, 1.64 mmol), tris(4-formylphenyl)amine (**3**, 0.360 g, 1.09 mmol), and dried THF (40 cm^3^). After dissolution of reagents, NaH was added under nitrogen cushion (0.164 g of 60% NaH in mineral oil, 4.1 mmol of pure compound). Reaction was stirred at room temperature overnight under dry nitrogen atmosphere and then refluxed under flowing nitrogen for 6 h. After cooling down, the suspension of dark yellow crude product in THF was transferred to tubes, thoroughly sonicated (10 min), and centrifuged at 10,000 RPM for 10 min. Next, crude product was redispersed in THF and was subjected to Soxhlet extraction with THF (25 cm^3^) for 6 h and then with methanol (25 cm^3^) for 6 h. Yellow precipitate was separated and dried. Yield 78% (0.472 g). Anal. Calcd (%) for C_39_H_27_N_4_: C, 84.91; H, 4.93; N, 10.16. Found: C, 82.78; H, 4.89; N, 9.70.

Synthesis of crosslinked conjugated polymer **Pol2**. In the flask was placed 4,4’-bis(diethoxyphosphorylmethyl)-2,2’-bipyridine (**1**, 0.348 g, 0.76 mmol), tris-4,4’,4’’-(4-formylphenylethynyl)triphenylamine (**5**, 0.320 g, 0.51 mmol), and dried THF (20 cm^3^). After dissolution of reagents, NaH was added under nitrogen cushion (0.076 g of 60% NaH in mineral oil, 1.9 mmol of pure compound). Reaction was stirred at room temperature overnight under dry nitrogen atmosphere and then refluxed under flowing nitrogen for 6 h. After cooling down, the suspension of dark yellow crude product in THF was transferred to tubes, thoroughly sonicated (10 min), and centrifuged at 10,000 RPM for 10 min. Next, crude product was redispersed in THF and was subjected to Soxhlet extraction with THF (25 cm^3^) for 6 h and then with methanol (25 cm^3^) for 6 h. Yellow precipitate was separated and dried. Yield 70% (0.303 g). Anal. Calcd (%) for C_63_H_39_N_4_: C, 88.81; H, 4.61; N, 6.58. Found: C, 86.70; H, 4.89; N, 6.13.

Synthesis of crosslinked conjugated polymer **Pol1-Eu**. In the flask was placed **Pol1** (0.100 g, 0.18 mmol of C_39_H_27_N_4_ repeating units, 0.27 mmol of 2,2’-bipyridine sites), europium tris(acetylacetonate) (0.485 g, 1.08 mmol), and methanol (25 cm^3^). Obtained suspension was refluxed for 48 h. After that time, reaction mixture was cooled down and transferred to tubes, thoroughly sonicated (10 min), and centrifuged at 10,000 RPM for 10 min. Supernatant was discarded, solids were redispersed in methanol (20 mL), sonicated, and centrifuged at 10,000 RPM for 10 min. Washing procedure was performed two more times. Yellow precipitate was separated and dried. ICP-OES analysis shows that the mass percentage of Eu^3+^ in **Pol1-Eu** is equal to 1.23%. Assuming that each Eu^3+^ preserves its tris(acetylacetonate) environment, C_39_H_27_N_4_·0.031(Eu(C_5_H_7_O_2_)_3_) formula can be proposed for **Pol1-Eu**. Yield: 80% (0.083 g). Anal. Calcd (%) for C_39.465_H_27.651_O_0.186_N_4_Eu_0.031_: C, 83.80; H, 4.92; N, 9.90. Found: C, 82.43; H, 4.71; N, 9.66.

### 3.3. Linear Optical Measurements

Fluorescence spectra were recorded with a fiber-coupled Ocean Optics QE-Pro FL spectrograph (Dunedin, FL, USA), upon excitation with a picosecond 377 nm BDL-377-SMC laser diode (Becker&Hickl, Berlin, Germany). The fluorescence kinetics was investigated with the use of the time-correlated single-photon counting (TCSPC) method. For TCSPC measurements, we used a Becker&Hickl system (Berlin, Germany) constructed from a TCSPC Module (SPC-130-EM) and a hybrid PMT detector (HPM-100-06) with detector control card (DCC 100) mounted to a spectrograph (Acton SpectraPro-2300i, Princeton Instruments, Trenton, NJ, USA). The sample was excited with a 377 nm BDL-377-SMC picosecond laser diode in the 20 MHz repetition mode (which corresponds to 50 ns collection time window). The fluorescence lifetime values were calculated, after deconvolution procedure of the instrument response function (IRF) which is ca. 200 ps.

### 3.4. Determination of Nonlinear Optical Properties With the Use of ISTPEF, SSTPEF and TPEF Techniques

NLO studies were performed using a femtosecond laser system comprising a Quantronix Integra-C regenerative amplifier (Quantronix Corp., East Setauket, NY, USA) operating as an 800 nm pump and a Quantronix-Palitra-FS optical parametric amplifier (Quantronix Corp., East Setauket, NY, USA). The latter was used to deliver wavelength tunable pulses (available range from 500 up to 2000 nm) with the duration < 130 fs, and a repetition rate of 1 kHz.

ISTPEF measurements: To the quartz cuvette of 1 cm optical path a known quantity of powder of polymer (**Pol1**, **Pol2**) was added (3–6 mg, amount adjusted to obtain strength of two-photon emission signal comparable in intensity to that of the two-photon reference). Next, 1 mL of 10^−5^ M Rhodamine B solution in methanol was added. The heterogeneous mixture of investigated polymers in Rhodamine B solution was stirred during measurements with the use of a magnetic stirrer at 500 rpm (Heidolph), and was excited with femtosecond laser in the range from 650 nm to 860 nm with 20 nm step. Note that intense stirring is crucial for temporal stability of the fluorescence signal. When necessary, the laser beam power was attenuated using a Glan laser polarizer (Thorlabs, Newton, NJ, USA). The fluorescence output from both components of the sample was collected in the backscattering geometry. Specifically, the beam was directed onto cuvette at about 45° whereas the collimator mounted to the glass optical fiber, was placed approximately in the perpendicular direction to the plane of the cuvette. The obtained fluorescence spectra were deconvoluted using two Gaussian functions per each fluorescence component with the use of OriginPro software (OriginLab, Northampton, MA, USA) in order to calculate integral intensities of contributions of polymer and of two-photon reference. In the case of excitation wavelengths lying closely to the emission spectrum of Rhodamine B a background subtraction procedure was necessary. Fluorescence spectra were recorded by a fiber-coupled Ocean Optics QE-Pro FL spectrograph (Dunedin, FL, USA). 

SSTPEF measurements: prior to SSTPEF measurements the solid samples of **Mod1**, **Mod2**, **Pol1** and **Pol2**, and that of reference compound BDPAS were sieved through a mini-sieve set (Sigma-Aldrich, St. Louis, MO, USA) to grain size smaller than 63 μm, and fixed between microscope glass slides. Glass slides were secured at both ends with transparent scotch tape and were excited in the range from 690 nm to 860 nm (20–25 nm step). Glass slides were horizontally placed on an adjustable holder. Next, the beam from the Palitra optical parametric amplifier was directed onto samples at about 45°, while the set of collimating lenses, mounted to the glass optical fiber, was placed perpendicular to the plane of the sample. One-photon excited fluorescence spectra were recorded with a fiber-coupled Ocean Optics QE-Pro FL spectrograph (Dunedin, FL, USA). TPEF measurements: 2PA cross sections were derived from the two-photon action cross-sections (σ_2_φ) and the fluorescence emission QY (φ). TPEF measurements were conducted relative to Rhodamine 6G in methanol using the method described by Xu and Webb [67]. Reference values of 2PA cross sections were taken from literature [58].

### 3.5. One-, Two-, and Three-Photon Luminescent Thermometry Study

A picosecond 377 nm BDL-377-SMC laser diode (Becker&Hickl, Berlin, Germany) was used as the one-photon excitation source for luminescence thermometric experiments on **Pol1-Eu**. The two- and three-photon excitation was provided by the amplified femtosecond system described above. The temperature control in the range 298–393K (with 3 K step) was performed with a thermostated heating plate (Heidolph, Schwabach, Germany) controlled by an external thermocouple attached to the sample. Luminescence spectra were recorded by a fiber-coupled Ocean Optics QE-Pro FL spectrograph (Dunedin, FL, USA).

## 4. Conclusions

In this contribution we have presented, for the first time, 2PA properties of crosslinked conjugated polymers, new members of an emerging class of NLO pigments. Polymers **Pol1** and **Pol2** were designed to accommodate three-dimensional crosslinked structure through double bond formation reaction between triphenylamine-based and 2,2’-bipyridine-based aromatic units, at the same time imparting the π-electron conjugation along the organic backbone. As is the case with other NLO pigments, the inherently aggregated, macromolecular structure of **Pol1** and **Pol2** causes that their 2PA properties could be investigated only in the solid state, particulate form. 

Spectrally-resolved 2PA properties of these solids were investigated by a combination of specially-modified TPEF-based techniques (ISTPEF, SSTPEF). We have found that self-aggregated structure of polymers **Pol1** and **Pol2** imparts two opposing effects that contribute to the observed strength of NLO response. The advantageous effect is that the intrinsic 2PA response of **Pol1** and **Pol2** is enhanced ca. 2-3 fold, when compared to **Mod1** and **Mod2** in solid state form. In addition to that, model compounds in solid state feature higher 2PA responses, compared to their chloroform solutions, thus highlighting the importance of aggregation phenomenon and its contribution to the enhancement of 2PA response in systems studied. Nevertheless, there is also deleterious effect associated with self-aggregation (ACQ phenomenon) which is responsible for relatively low QYs of fluorescence and significantly shortened fluorescence lifetimes of **Pol1** and **Pol2**. The analysis of molar mass-normalized two-photon brightness values (σ_2_φ/M) shows that the net effect is negative, i.e., the benefit of higher 2PA responses (σ_2_, σ_2_/M) is canceled out and even overwhelmed by decreased QYs. Accordingly, future studies on crosslinked conjugated polymers should focus not only on the maximalization of the intrinsic 2PA cross section values, but also on preserving as large as possible quantum efficiency of fluorescence, thus maximizing the two-photon brightness of the material. One direction that will be pursued in the future is the incorporation into the polymer structure organic fragments that feature the aggregation-induced emission (AIE) phenomenon.

We also explored what are the perspectives for **Pol1** and **Pol2** to be used in linear and nonlinear optical remote thermometry applications. Since those materials do not offer emission bands of disparate thermal quenching behavior, we prepared a postfunctionalized material by simple complexation reaction of the crosslinked conjugated polymer **Pol1** with a source of Eu^3+^ ions (**Pol1-Eu**). We found that the emission spectrum of **Pol1-Eu** indeed contains combination of organic backbone emission and Eu^3+^-centered luminescence and that these contributions feature different thermal quenching behavior. We demonstrated that temperature-dependent emissions can be excited not only upon linear excitation at 377 nm, but also signals with good signal-to-noise intensity can be obtained by using 2PA and 3PA-mediated excitation modes at 820 nm and 1020 nm, respectively. The collected data show that **Pol1-Eu** is responsive to temperature changes, yet the observed temperature characteristics of thermometric parameter values are not the same for heating and cooling runs, irrespectively of the excitation mode used, i.e., the ratio of integral intensities of polymer-centered and Eu^3+^-centered emission is different before and after heating-cooling cycle. Moreover, **Pol1-Eu** is found to be sensitive to prolonged laser irradiation. Accordingly, **Pol1-Eu** is of little use for real thermometric applications, but it is the first example of a polymeric material for which responsiveness of 2PA- and 3PA-excited luminescence to temperature has been demonstrated.

## Figures and Tables

**Table 1:** Collection of linear and nonlinear optical parameters for **Mod1**, **Mod2**, **Pol1**, and **Pol2** determined using solid state TPEF (SSTPEF), ISTPEF, and TPEF techniques.

	Linear Optical Parameters			Nonlinear Optical Parameters				
Material	λ_max_ λ_exc._ = 377 nm	τ (ns) at λ_max_	φ λ_exc._ = 377 nm	Technique	σ_2_ (GM), λ ^c^	σ_2_·φ	σ_2_/M	σ_2_·φ/M
**Mod1**	504 nm, CHCl_3_	1.98 ^a^	0.54	TPEF	219, 690 nm	118	0.34	0.18
	540 nm, s.s.	1.92 ^b^	0.32	SSTPEF	390, 700 nm	125	0.56	0.18
**Mod2**	523 nm, CHCl_3_	2.04 ^a^	0.79	TPEF	502, 780 nm	396	0.56	0.44
	485, 523 nm, s.s.	1.70 ^b^	0.16	SSTPEF	982, 700 nm	157	1.10	0.18
**Pol1**	501 nm, s.s.	0.79 ^b^	0.11	ISTPEF	576, 690 nm	63	0.96	0.11
				SSTPEF	850, 690 nm	93	1.42	0.16
**Pol2**	490 nm, s.s.	0.77 ^b^	0.07	ISTPEF	839, 700 nm	58	0.94	0.07
				SSTPEF	1494, 700 nm	104	1.67	0.12

s.s.: solid state, ^a^ monoexponential decay, ^b^ mean fluorescence lifetime calculated using triexponential model, ^c^ relative error of σ_2_ values was estimated to be around 15%.

## Supplementary Materials

Supplementary materials are available online at https://www.mdpi.com/2073-4360/12/8/1670/s1.
