# Supplementary material for: Nonlinear Optical Pigments. Two-Photon Absorption in Crosslinked Conjugated Polymers and Prospects for Remote Nonlinear Optical Thermometry
Source: Polymers (Basel). 2020 Jul 27;12(8):1670. doi: 10.3390/polym12081670 (PMC7463668; doi:10.3390/polym12081670)
Supplement: Supplementary file 1 [file polymers-12-01670-s001.pdf]

Supporting Information to:

Nonlinear optical pigments. Two-photon absorption in crosslinked conjugated polymers and perspectives of remote NLO thermometry

Jan K. Zaręba[1,*], Marcin Nyk[1] and Marek Samoć[1]

[1] Advanced Materials Engineering and Modelling Group, Wroclaw University of Science and Technology, Wyb. Wyspiańskiego 27, 50370, Wrocław, Poland; marcin.nyk@pwr.edu.pl (M.N.), marek.samoc@pwr.edu.pl (M.S.)
* Correspondence: jan.zareba@pwr.edu.pl (J.Z.).

1. Synthetic procedures

Materials

Starting materials were of reagent grade purity and were obtained from commercial sources (Aldrich and Avantor Chemicals, Poland). THF, toluene, dioxane and methanol used for syntheses were kept over 3 Å molecular sieves.

Synthetic path to 4,4′-bis(diethoxyphosphorylmethyl)-2,2′-bipyridine, (1)

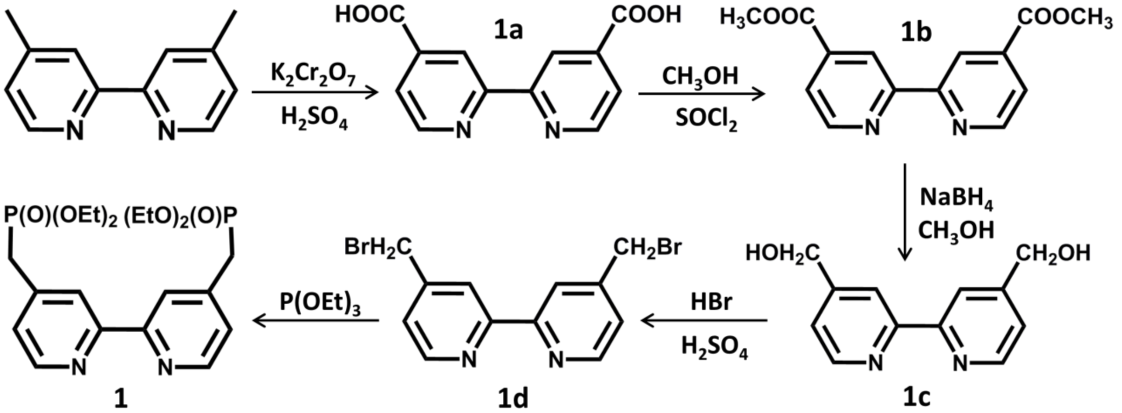

Synthesis of 2,2'-bipyridine-4,4'-dicarboxylic acid, (1a)

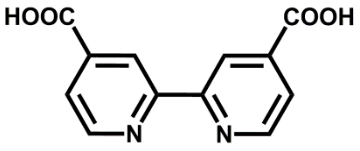

In a two-neck flask was placed 4,4-dimethyl-2,2-bipyridine (4.000 g, 21.7 mmol) and 98% sulfuric acid (70 cm^3) was carefully added dropwise. After cooling to to room temperature, potassium dichromate (3.3g, 11.3mmol) was added in portions (the solution color turns dark green). Both processes are strongly exothermic; hence, in each case, the reaction flask is immersed in water bath for better dissipation of produced heat. Next, the reaction mixture was heated to 65 °C and was maintained at that temperature for 6 h under stirring. After that time, reaction mixture was cooled to room temperature, the reaction mixture poured into 500 cm^3 of water, forming greenish precipitate which was filtered out under reduced pressure, and washed with a small amount of water. Next, crude compound was dissolved in a minimal amount of ammonia affording dark-colored solution which was filtered through paper filter (chromium-containing precipitate was discarded), and the filtrate was acidified with 1M HCl to obtain suspension of 2,2'-bipyridine-4,4'-dicarboxylic acid. It was filtered out under reduced pressure, washed with small amount of water, and dried. Pure 2,2'-Bipyridine-4,4'-dicarboxylic acid was obtained (4.811g, 91%).
^{1}H NMR (400 MHz, D_2O+NaOD) δ 8.64 (2H, s), 8.23 (2H, s), 7.75 (2H, s). Note that peaks appear as singlets due to proton-exchange broadening.

Synthesis of 4,4'-bis(carbomethoxy)-2,2'-bipyridine, (1b)

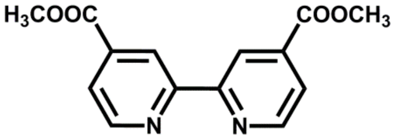

In two-neck flask were placed 2,2'-Bipyridine-4,4'-dicarboxylic acid (**1a**, 4.000 g, 16.4 mmol) and anhydrous methanol (100 cm^3), forming a white suspension. Next, under intense stirring of reaction mixture thionyl chloride (5 cm^3, 69.0 mmol) was carefully added in a dropwise fashion. Next, the mixture was heated at reflux overnight. After cooling, solvent was evaporated under reduced pressure and the obtained white solid was dissolved in chloroform (50 cm^3). The obtained solution was transferred to the separation funnel, washed with 10% Na_2CO_3 aqueous solution (50 cm^3), once with water (50 cm^3), once with NaCl saturated solution (50 cm^3), and finally dried with anhydrous Na_2SO_4. Evaporation of organic solvents and crystallization from ethyl acetate afforded pure 4,4'-bis(carbomethoxy)-2,2'-bipyridine (3.790 g, 85%).

^{1}H NMR (400 MHz, $CDCl_3$, 300K) δ 8.95 (2H, s), 8.85 (2H, d, *J* = 4.8 Hz), 7.89 (2H, dd, *J* = 4.9, 1.4 Hz), 3.99 (6H, s).

Synthesis of 4,4'-bis(hydroxymethyl)-2,2'-bipyridine, (1c)

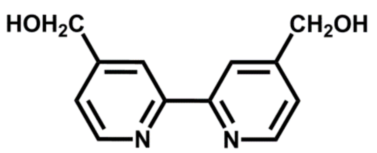

In a two-neck flask were placed 4,4'-bis(carbomethoxy)-2,2'-bipyridine (**1b**, 3.500 g, 12.8 mmol), $NaBH_4$ (3 g, 79.3 mmol), and ethanol (50 cm^3). The mixture was refluxed for 4 h. After cooling to room temperature, saturated solution of NH_4Cl (100 cm^3) was added to the mixture, which was stirred for 15 min. The ethanol was removed under vacuum and the resulting white precipitate was dissolved in the minimum quantity of water. The solution was extracted with chloroform (5×50 cm^3) and the combined organic fractions were washed witch brine, and then dried with Na_2SO_4. After evaporation of solvent, pure 4,4'-bis(hydroxymethyl)-2,2'-bipyridine is obtained as white solid (1.700 g, 61%).

^{1}H NMR (400 MHz, $CDCl_3$, 300K) δ 8.67 (2H, d, *J* = 5.0 Hz), 8.39 (2H, s), 7.37 (2H, d, *J* = 4.9 Hz), 4.85 (4H, d, *J* = 5.9 Hz), 1.89 (2H, t, *J* = 6.1 Hz).

Synthesis of 4,4′-bis(bromomethyl)-2,2′-bipyridine, (1d)

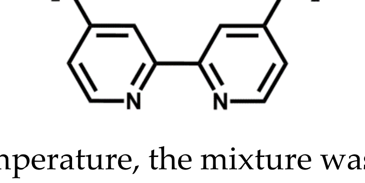

In a two-neck flask were placed 4,4'-bis(hydroxymethyl)-2,2'-bipyridine (**1c**, 2.770 g, 9.25 mmol) dissolved in 48 % HBr_{aq} (30 cm^3) and 98 % H_2SO_4 (12 cm^3). Obtained solution was refluxed for 12h. After cooling to room temperature, the mixture was neutralized with solid CH_3COONa. The resulting white precipitate was filtered off and dried. The solid was then dissolved in ethyl acetate (20 cm^3), obtained solution was dried over Na_2SO_4, and passed through a short plug of silica. No further purification was required. After evaporation of solvent, pure 4,4′-

bis(bromomethyl)-2,2′-bipyridine was obtained as yellowish solid (1.754 g, 88%). *Caution! This compound has lacrimatory properties.*

^{1}H NMR (400 MHz, $CDCl_3$, 300K) δ 8.68 (2H, d, *J* = 5.0 Hz), 8.45 (2H, d, *J* = 1.2 Hz), 7.37 (2H, dd, *J* = 5.0, 1.8 Hz, 2H), 4.49 (4H, s).

Synthesis of 4,4′-bis(diethoxyphosphorylmethyl)-2,2′-bipyridine, (1)

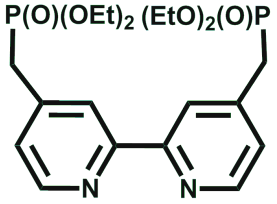

In a three-neck flask fitted with a volatiles trap were placed 4,4′-bis(bromomethyl)-2,2′-bipyridine (**1d**, 2.00 g, 5.85 mmol) and triethyl phosphite (8.0 cm^3, 46.7 mmol). The mixture was heated to 120 °C under constant flow of nitrogen in order to remove ethyl bromide from the reaction system. After 3 hours, the volatiles were distilled off under reduced pressure at the same temperature. Orange-colored crude product was purified by silica-gel column chromatography (eluent consisting of ethyl acetate/MeOH/trimethylamine, 90:9:1 by volume). Pure 4,4′-bis(diethoxyphosphorylmethyl)-2,2′-bipyridine (2.370 g, 89%) is obtained as an oily compound that rapidly crystallizes as a white solid.

^{1}H NMR (400 MHz, $CDCl_3$, 300K) δ 8.60 (2H, d, *J* = 5.0 Hz), 8.34 (2H, s), 7.35 – 7.28 (2H, m), 4.29 – 3.83 (8H, m), 3.23 (4H, d, *J* = 22.2 Hz), 1.26 (12H, t, *J* = 7.0 Hz). ^{31}P NMR (162 MHz, $CDCl_3$, 300K) δ 24.26 (s).

Synthetic paths to 4-formyltriphenylamine (2) and tris(4-formylphenyl)amine (3)

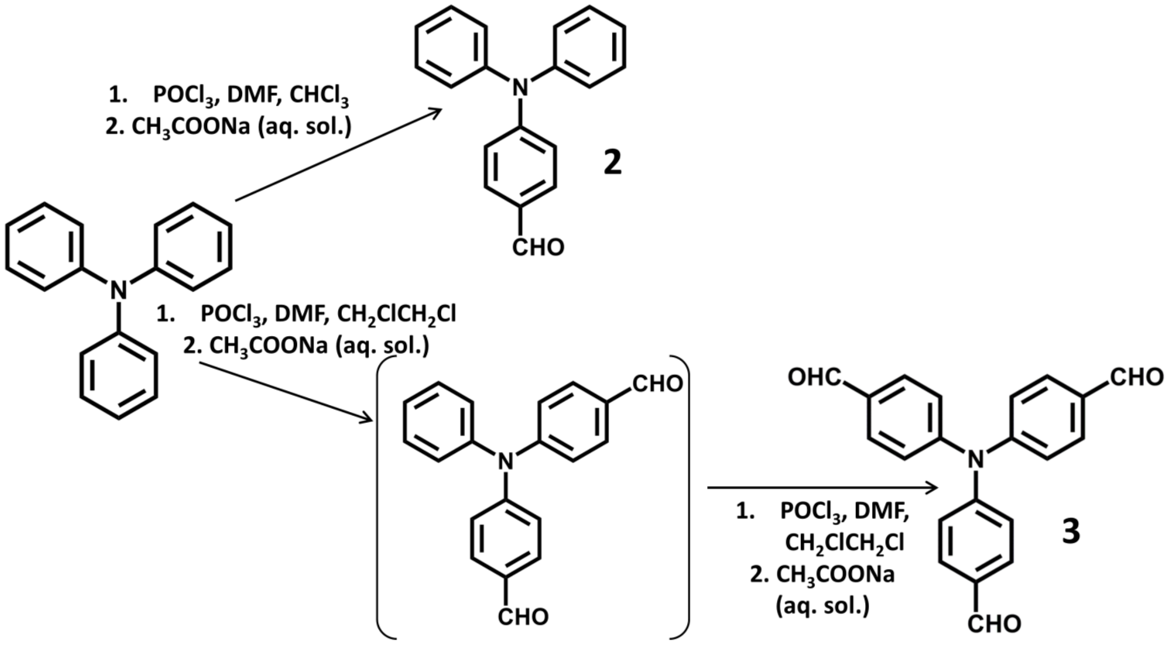

Synthesis of 4-formyltriphenylamine, (2)

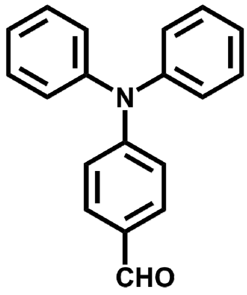

Vilsmeier–Haack formylation reaction was employed to obtain 4-formyltriphenylamine. In a two-neck flask was placed DMF (6.31 cm^3, 81.5 mmol) Next, $POCl_3$ (8.57 cm^3, 91.7 mmol), under the atmosphere of nitrogen, was added in small portions at 0 °C. The mixture was stirred until the glassy solid was obtained. Next, a solution of triphenylamine (5.00 g, 20.4 mmol) in chloroform (50 cm^3) was added. Reaction mixture was refluxed for 6 hours and then cooled to room temperature. After cooling, the mixture was poured in portions into the 200 cm^3 of 20% aqueous solution of sodium acetate. Biphasic mixture was stirred until organic layer changed color to yellow. Organic layer was separated. Organic extract was washed with water (50 cm^3), two times with NaCl saturated solution (2 x 50 cm^3), and finally dried with anhydrous Na_2SO_4. A pale yellow solid was collected by evaporation of organic solvents and recrystallized from ethanol to give pure 4-formyltriphenylamine (4.20 g, 75%).

^{1}H NMR ($CDCl_3$ 400 MHz, 300K) δ 9.80 (1H, s), 7.67 (2H, d, J = 8.9 Hz), 7.38 – 7.30 (4H, m), 7.19 – 7.14 (6H, m), 7.00 (2H, d, J = 8.8 Hz).

Synthesis of tris(4-formylphenyl)amine, (3)

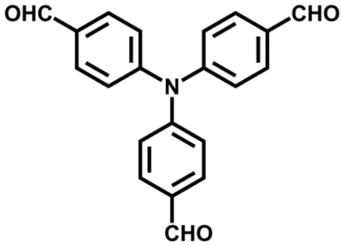

A modified two-step Vilsmeier–Haack Formylation reaction was employed to obtain tris(4-formylphenyl)amine. This protocol rests on synthesis of bis(4-formylphenyl)aniline in the first synthetic step, followed by additional formylation reaction in the second step. In two-neck flask was placed DMF (14.5 cm^3, 187.4 mmol) and $POCl_3$ (20.00 cm^3, 214.6 mmol) was added in small portions at 0 °C under the atmosphere of nitrogen. The mixture was stirred till the glassy solid was obtained. Next, triphenylamine (2.50 g, 10.2 mmol) was added. Reaction mixture was kept at 95 deg C. for 4h under nitrogen and then cooled to room temperature. After cooling, glassy solid was dissolved in chloroform and the mixture was poured in portions into the 300 cm^3 of 20% aqueous solution of sodium acetate. Biphasic mixture was stirred until organic layer changed color to orange. The organic layer was separated and was washed with water (50 cm^3), two times with NaCl saturated solution (2 x 50 cm^3) and finally dried with anhydrous Na_2SO_4. Crude bis(4-formylphenyl)aniline was dissolved in a minimal amount of chloroform was passed through short layer of silica gel in order to remove dark-colored impurities. No additional purifications of crude intermediate product were performed, and in the second step the crude bis(4-formylphenyl)aniline was subjected to additional formylation. In a two-neck flask was placed DMF (14.51 cm^3, 187.4 mmol) and $POCl_3$ (20.05 cm^3, 214.6 mmol) was added in small portions at 0 °C under the atmosphere of nitrogen. The mixture was stirred until the glassy solid was obtained. Next, a powdered crude bis(4-formylphenyl)aniline was added. Reaction mixture was kept at 95 deg C. for 2h under nitrogen and then cooled to room temperature. After cooling, glassy solid was dissolved in chloroform and the mixture was poured in portions into the 300 cm^3 of 20% aqueous solution of sodium acetate. Organic layer was separated and was washed with water (50 cm^3), two times with NaCl saturated solution (2 x 50 cm^3), and finally dried with anhydrous Na_2SO_4. After evaporation yellow solid was subjected to column chromatography (dichloromethane) to give pure tris(4-formylphenyl)amine (1.609 g, 48%).

^{1}H NMR ($CDCl_3$, 400 MHz, 300K) δ 9.95 (3H, s), 7.84 (6H , d, J= 8.5 Hz), 7.25 (6H, d, J= 8.3 Hz).

Synthetic path to 4-((4-(diphenylamino)phenyl)ethynyl)benzaldehyde, (4)

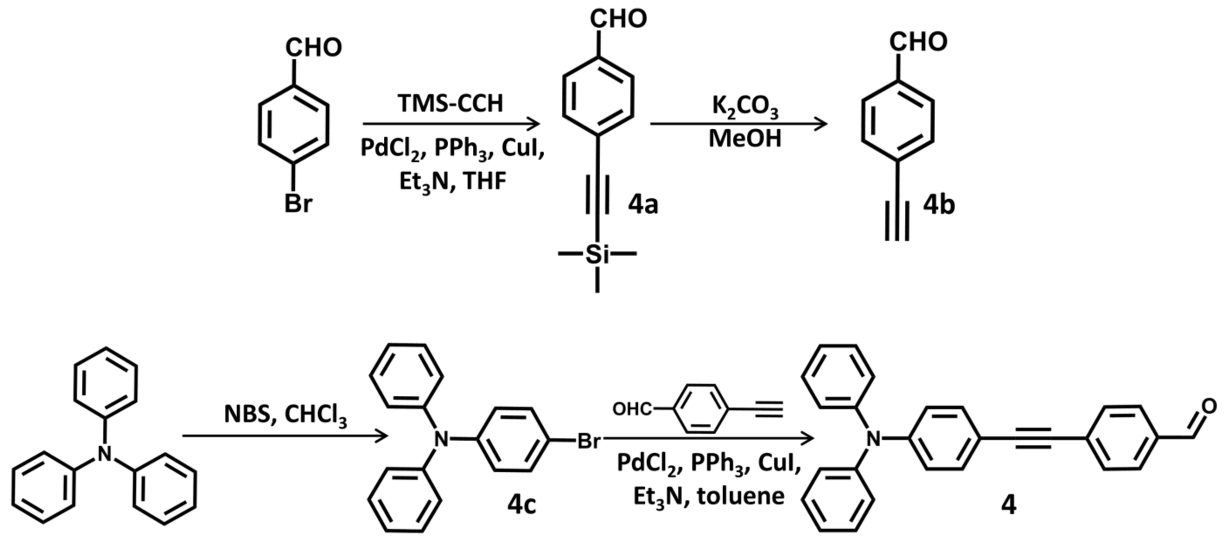

Synthesis of 4-trimethylsilylethynylbenzaldehyde, (4a)

In a two-neck flask were placed 4-bromobenzaldehyde (5.00 g, 27.0 mmol), CuI (0.256 g, 1.34 mmol), $PdCl_2$ (0.125g, 0.70 mmol), triphenylphosphine (0.737 g, 2.81 mmol), and 25 cm^3 of THF. After purging with nitrogen, triethylamine (11.3 cm^3, 81.0 mmol), and trimethylsilylacetylene (5.38 cm^3, 37.8 mmol) were added. The mixture was stirred for 3 hours at reflux, then poured into excess of water and extracted with $CHCl_3$. The organic layer was separated and was washed with water (50 cm^3), two times with NaCl saturated solution (2 x 50 cm^3), dried with anhydrous Na_2SO_4, and filtered. The filtrate was evaporated. The obtained crude compound was subjected to column chromatography (eluent hexane: $CHCl_3$ = 15:1 by volume) to give pure 4-trimethylsilylethynylbenzaldehyde (4.890 g, 90%) as a brownish solid.

^{1}H NMR ($CDCl_3$ 400 MHz, 300K) δ 10.01 (1H, s), 7.83 (2H, dd, J = 6.6, 1.8 Hz), 7.61 (2H, d, J = 8.3 Hz), 0.27 (9H, s)

Synthesis of 4-Ethynylbenzaldehyde, (4b)

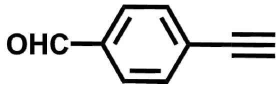

In a two-neck flask were placed 4-trimethylsilylethynylbenzaldehyde (**4a**, 3.000 g, 14.8 mmol), K_2CO_3 (0.410 g, 2.96 mmol) and 30 cm^3 of methanol. The mixture was stirred at room temperature until its complete substrate conversion into deprotected alkyne (monitored using TLC, ca. 2 h). The solvent was evaporated, and the obtained solid was dissolved in 50 cm^3 of chloroform. The organic layer was washed with water (30 cm^3), once with NaCl saturated solution (30 cm^3), dried with

anhydrous Na_2SO_4, and filtered. The filtrate was evaporated. Pure 4-ethynylbenzaldehyde (1.802 g, 93%) was obtained as a yellow solid.

^{1}H NMR (400 MHz, $CDCl_3$, 300K) δ 10.00 (1H, s), 7.82 (2H, d, *J* = 7.5 Hz), 7.62 (2H, d, *J* = 7.8 Hz), 3.28 (1H, s).

Synthesis of 4-*N,N*-diphenylamino-1-bromobenzene, (4c)

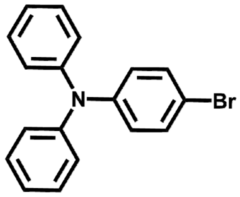

In a two-neck flask were placed triphenylamine (9.210 g, 37.50 mmol) and N-Bromosuccinimide (NBS, 6.680 g, 37.50 mmol) were dissolved in 120 mL carbon tetrachloride and obtained solution was refluxed for 5 h. After that time reaction mixture was cooled to room temperature. Precipitate (succinimide) was filtered out and discarded. Filtrate was evaporated, and obtained oil was crystallized from ethanol once. Pure 4-*N,N*-diphenylamino-1-bromobenzene was obtained as fine white needles (9.595 g, 79%).

^{1}H NMR (400 MHz, $CDCl_3$, 300K) δ 7.35 (2H, d, *J* = 8.6 Hz), 7.28 (4H, t, *J* = 7.7 Hz), 7.10 (4H, d, *J* = 8.3 Hz), 7.06 (2H, t, *J* = 7.4 Hz), 6.97 (2H, d, *J* = 8.6 Hz).

Synthesis of 4-((4-(diphenylamino)phenyl)ethynyl)benzaldehyde, (4)

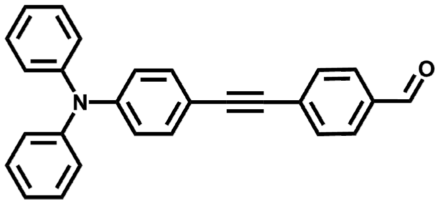

In a two-neck flask were placed 4-*N,N*-diphenylamino-1-bromobenzene (**4c**, 5.00 g, 27.0 mmol), CuI (0.256 g, 1.34 mmol), $PdCl_2$ (0.125g, 0.70 mmol), triphenylphosphine (0.737 g, 2.81 mmol), triethylamine (11.3 cm^3, 81.0 mmol), and 25 cm^3 of toluene under continuous flow of nitrogen. Next, 4-ethynylbenzaldehyde (**4b**, 5.38 g, 37.8 mmol) was added. The mixture was stirred overnight at 75 °C. After cooling, the toluene was evaporated under reduced pressure. Next, the sticky solid was dissolved in $CHCl_3$ (50 cm^3). Organic layer was washed with water (50 cm^3), once with NaCl saturated solution (50 cm^3), dried with anhydrous Na_2SO_4, and filtered. The filtrate was evaporated. The obtained crude compound was subjected to column chromatography (eluent hexane:$CHCl_3$ = 1:1 by volume) on silica gel to give pure 4-((4-(diphenylamino)phenyl)ethynyl)benzaldehyde (4.890 g, 90%) as a pale yellow solid.

^{1}H NMR (400 MHz, $CDCl_3$, 300K) δ 10.01 (1H, s), 7.85 (2H, d, *J* = 8.3 Hz), 7.64 (2H, d, *J* = 8.2 Hz), 7.39 (2H, d, *J* = 8.7 Hz), 7.29 (4H, t, *J* = 7.9 Hz), 7.16 – 7.06 (6H, m), 7.01 (2H, d, *J* = 8.7 Hz).

Synthetic path to tris-4,4',4''-(4-formylphenylethynyl)triphenylamine

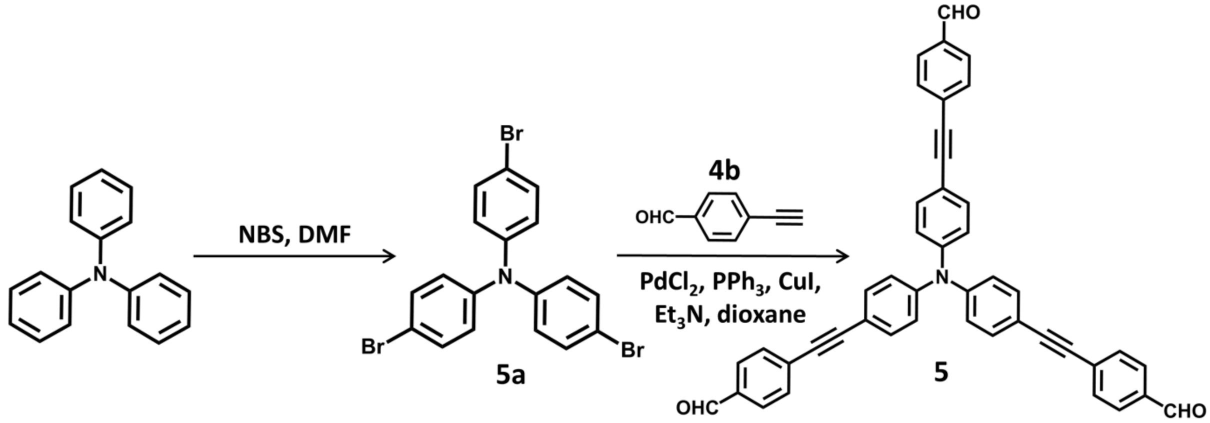

Synthesis of Tris(4-bromophenyl)amine, (5a)

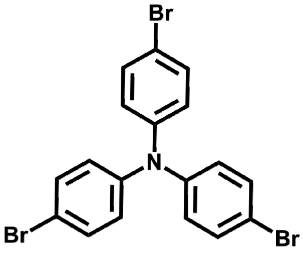

In a two-neck flask were placed triphenylamine (3.000 g, 12.2 mmol) and DMF (50 cm^3). Next, it was immersed in an ice-bath to cool to the solution to ca. 0 °C. To the obtained solution, under continuous stirring NBS (7.180 g, 40.3 mmol) dissolved in DMF (20 cm^3) was added dropwise. After complete addition, the solution mixture was warmed up to room temperature and left overnight. Next day, the reaction mixture was poured into water (300 cm^3). Obtained light gray precipitate was filtered out under reduced pressure and washed with copious amounts of water in order to wash out remnants of DMF and succinimide. Crude product was crystallized from ethanol once. Pure tris(4-bromophenyl)amine was obtained as white needles (4.580 g, 78%).

^{1}H NMR (400MHz, $CDCl_3$, 300K) δ 7.35 (6H, dd, J= 6.6, 2.0 Hz), 6.92 (6H, dd, J= 6.6, 2.1 Hz).

Synthesis of tris-4,4',4''-(4-formylphenylethynyl)triphenylamine, (5)

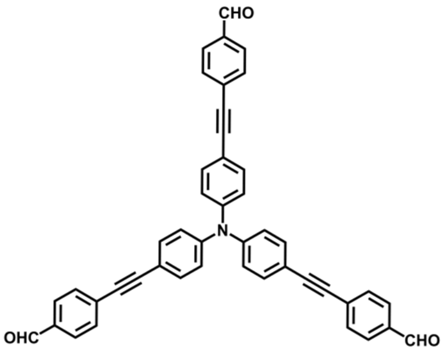

In a two-neck flask were placed tris(4-bromophenyl)amine (**5a**, 1.00 g, 2.07 mmol), CuI (0.059 g, 0.31 mmol), $PdCl_2$ (0.028 g, 0.155 mmol), triphenylphosphine (0.079 g, 0.30 mmol), triethylamine (2.6 cm^3, 18.6 mmol), and 20 cm^3 of dioxane under continuous flow of nitrogen. Next, 4-ethynylbenzaldehyde (**4b**, 1.470 g, 10.3 mmol) was added. The mixture was stirred for 48h at 75 °C. After cooling, dioxane was evaporated under reduced pressure. Next, the sticky solid was dissolved in $CHCl_3$ (30 cm^3). Organic layer was washed with water (30 cm^3), once with NaCl saturated solution (30 cm^3), dried with anhydrous Na_2SO_4, and filtered. The

filtrate was evaporated. The obtained crude compound was subjected to column chromatography (chloroform) on silica gel to give pure tris-4,4',4''-(4-formylphenylethynyl)triphenylamine (0.435 g, 33%) as flaky pale yellow solid.

^{1}H NMR (400MHz, $CDCl_3$, 300K) δ 10.02 (3H, s), 7.87 (6H, d, *J* = 8.5 Hz), 7.66 (6H, d, *J* = 8.2 Hz), 7.48 (6H, d, *J* = 8.8 Hz), 7.11 (6H, d, *J* = 8.8 Hz).

2. Supplementary figures

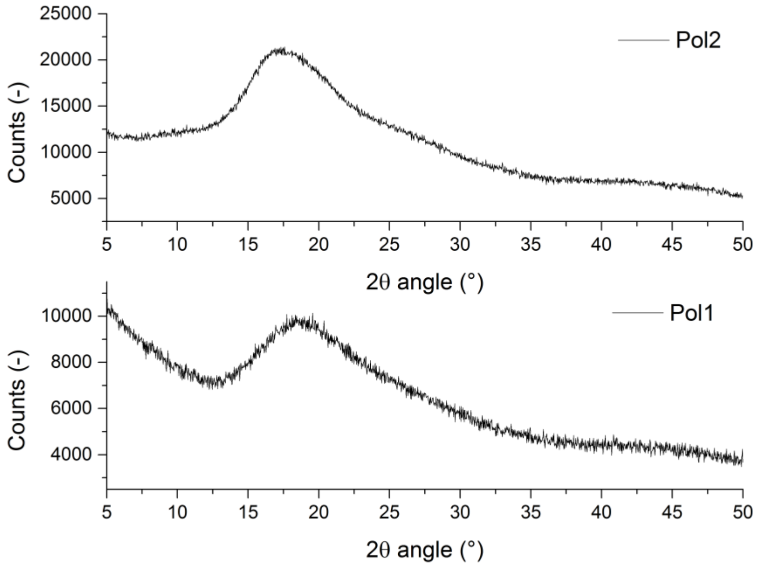

Figure S1. Experimental PXRD pattterns obtained for **Pol1** (lower panel) and **Pol2** (upper panel).

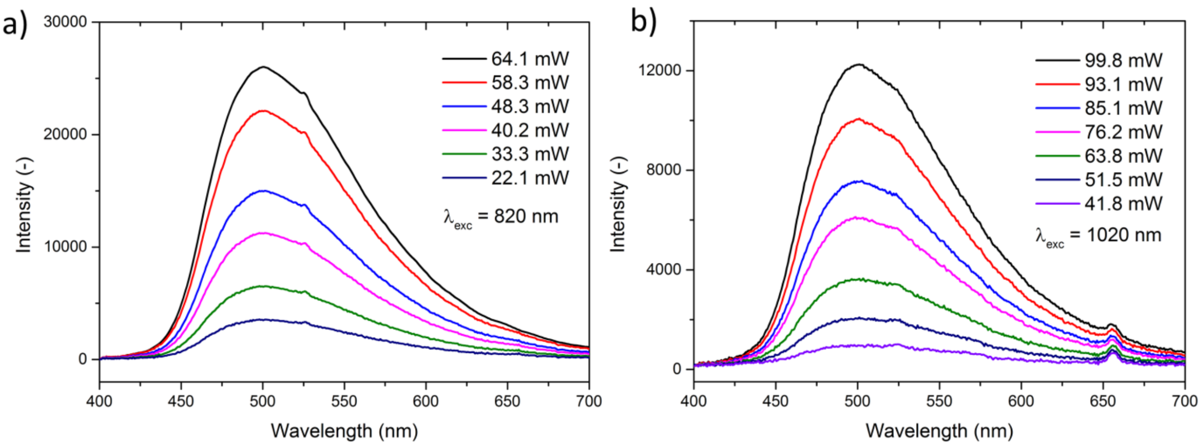

Figure S2. Emission spectra of **Pol1** measured in the function of the applied laser power at a) λ_{exc} = 720 nm, b) λ_{exc} = 1020 nm.

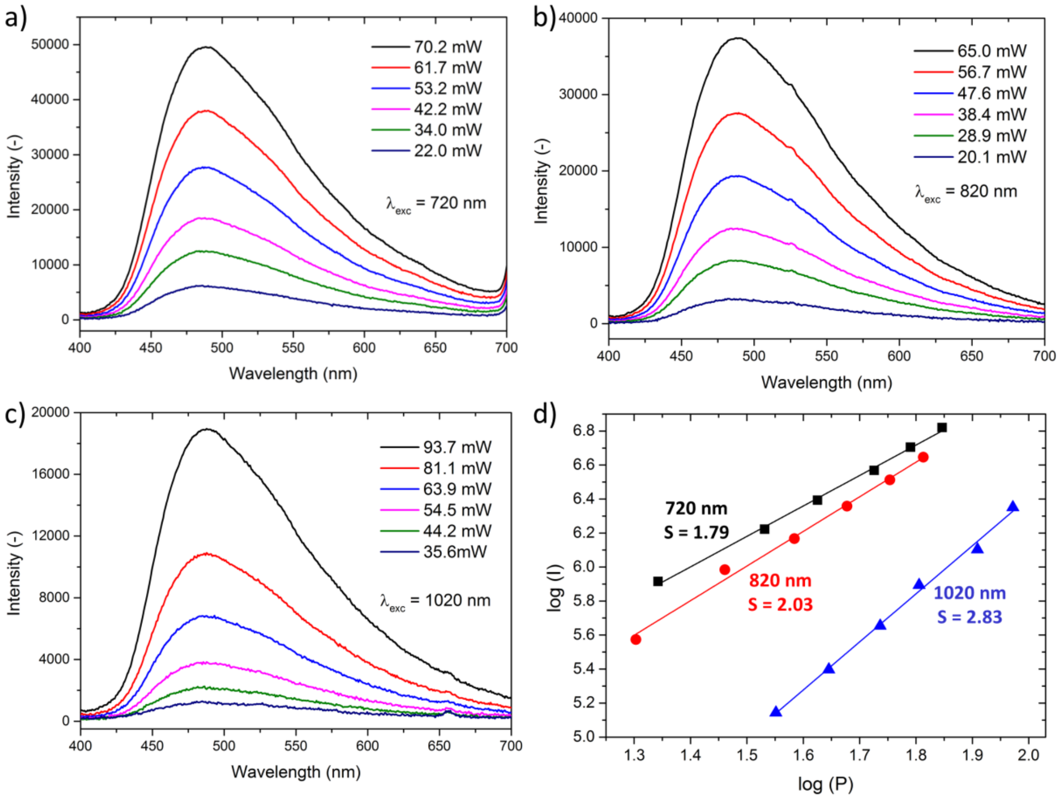

Figure S3. Emission spectra of **Pol2** measured in the function of the applied laser power at a) λ_{exc} = 720 nm, b) λ_{exc} = 820 nm, c) λ_{exc} = 1020 nm. d) Log–log plots of integral intensities of **Pol1** emissions excited at 720 nm, 820 nm and 1020 nm in the function of applied laser power. Slopes indicate 2PA as the origin of emissions at 720 nm and 820 nm and 3PA at 1020 nm.

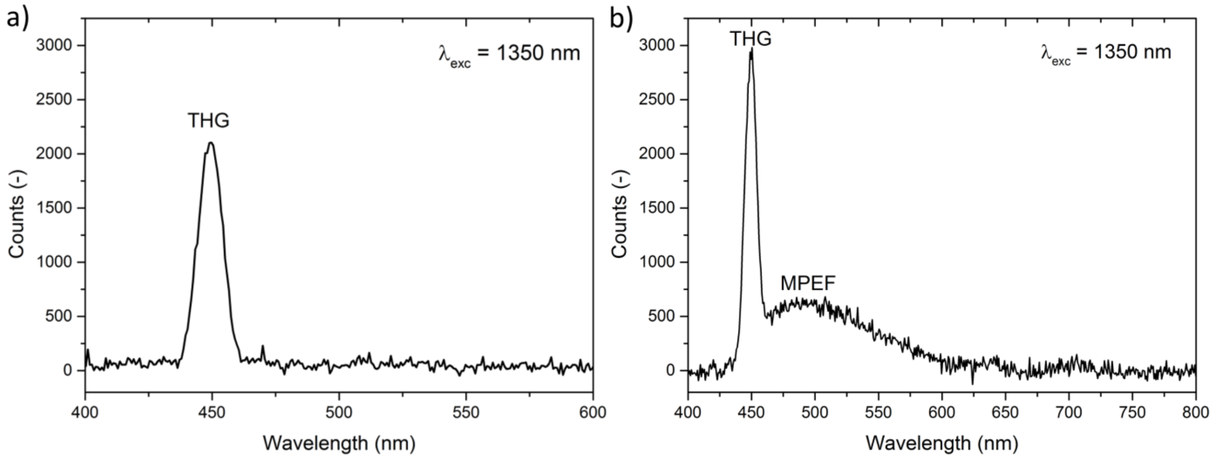

Figure S4. Spectrum obtained upon irradiation of a) **Pol1,** b) **Pol2** with 1350 nm femtosecond laser pulses (P=80mW). MPEF stands for multi-photon excited fluorescence.

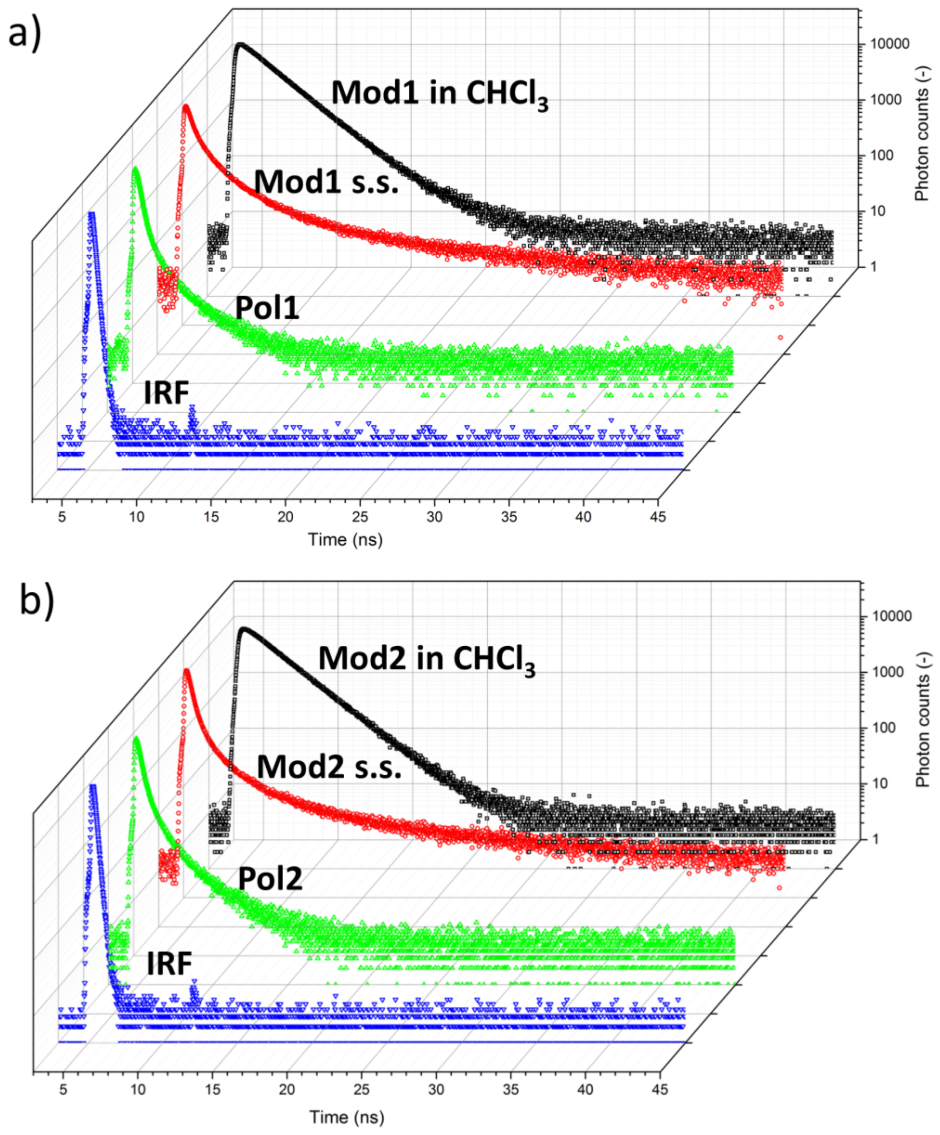

Figure S5. Fluorescence decay curves of a) **Mod1** in $CHCl_3$**, Mod1** as solid state (s.s.), **Pol1,** instrument response function (IRF) and b) **Mod2** in $CHCl_3$, **Mod2** as solid state, **Pol2** and IRF. Decay data for both **Mod1** and **Mod2** in $CHCl_3$ solutions were satisfactorily described by monoexponential functions, while decay data for all solid state samples were best described by triexponential function. Decay times were calculated from weighted average $\tau_{mean} = (A_1 \cdot \tau_1 + A_2 \cdot \tau_2 + A_2 \cdot \tau_3)/(A_1 + A_2 + A_3)$.

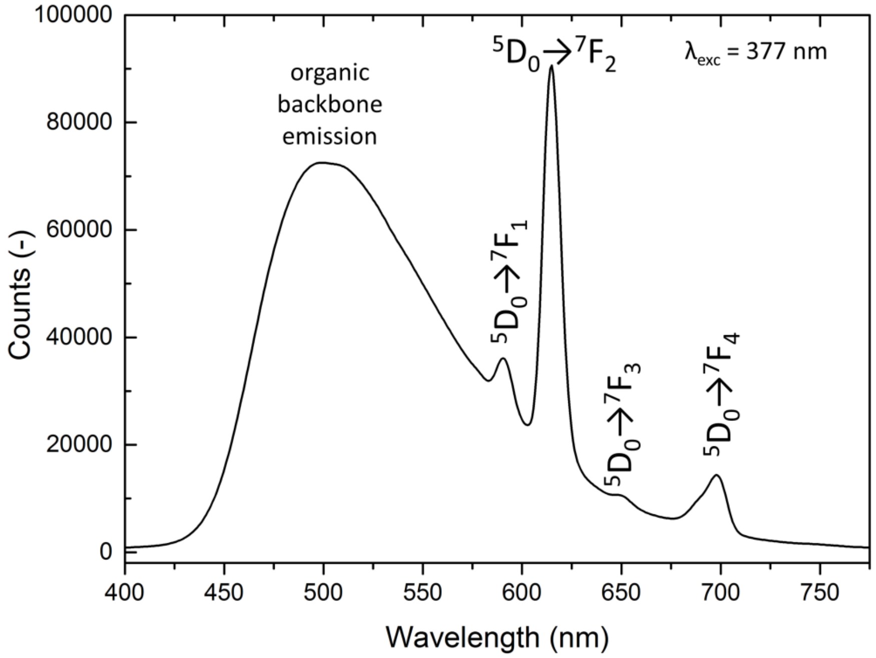

Figure S6. Emission spectra of **Pol1-Eu** ($\lambda_{exc.}$ = 377 nm) measured at 298K.

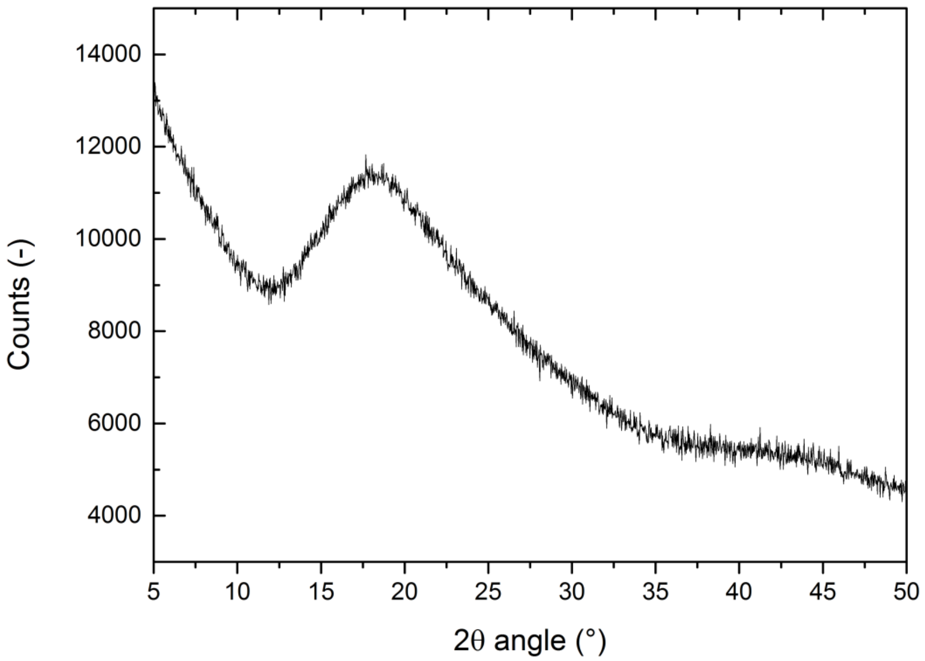

Figure S7. Experimental PXRD pattttern obtained for **Pol1-Eu.**
